# Intermittent F-actin Perturbations by Magnetic Fields Inhibit Breast Cancer Metastasis

**DOI:** 10.34133/research.0080

**Published:** 2023-03-15

**Authors:** Xinmiao Ji, Xiaofei Tian, Shuang Feng, Lei Zhang, Junjun Wang, Ruowen Guo, Yiming Zhu, Xin Yu, Yongsen Zhang, Haifeng Du, Vitalii Zablotskii, Xin Zhang

**Affiliations:** ^1^ High Magnetic Field Laboratory of CAS (CHMFL), CAS Key Laboratory of High Magnetic Field and Ion Beam Physical Biology, HFIPS, Hefei, Anhui 230031, P.R China.; ^2^ Institutes of Physical Science and Information Technology, Anhui University, Hefei, Anhui, 230601, P. R. China.; ^3^ Science Island Branch of Graduate School, University of Science and Technology of China, Hefei, Anhui 230031, P.R China.; ^4^ Institute of Physics of the Czech Academy of Sciences, Prague, Czech Republic.; ^5^ International Magnetobiology Frontier Research Center, Science Island, Hefei 230031, P.R. China.

## Abstract

F-actin (filamentous actin) has been shown to be sensitive to mechanical stimuli and play critical roles in cell attachment, migration, and cancer metastasis, but there are very limited ways to perturb F-actin dynamics with low cell toxicity. Magnetic field is a noninvasive and reversible physical tool that can easily penetrate cells and human bodies. Here, we show that 0.1/0.4-T 4.2-Hz moderate-intensity low-frequency rotating magnetic field-induced electric field could directly decrease F-actin formation in vitro and in vivo, which results in decreased breast cancer cell migration, invasion, and attachment. Moreover, low-frequency rotating magnetic fields generated significantly different effects on F-actin in breast cancer vs. noncancerous cells, including F-actin number and their recovery after magnetic field retrieval. Using an intermittent treatment modality, low-frequency rotating magnetic fields could significantly reduce mouse breast cancer metastasis, prolong mouse survival by 31.5 to 46.0% (*P* < 0.0001), and improve their overall physical condition. Therefore, our work demonstrates that low-frequency rotating magnetic fields not only can be used as a research tool to perturb F-actin but also can inhibit breast cancer metastasis through F-actin modulation while having minimum effects on normal cells, which reveals their potential to be developed as temporal-controlled, noninvasive, and high-penetration physical treatments for metastatic cancer.

## Introduction

Cytoskeleton is a fibrous network of protein filaments, including microtubules, actin, and intermediate filaments, which are not only responsible for cell morphology maintenance and organelle position but also key components for multiple cellular processes, including membrane trafficking, signal transduction, and cell migration and division.

As one of the major components of cytoskeleton, actin is the most abundant cellular protein in most types of eukaryote cells. G-actin (globular actin) monomers in cells can polymerize into F-actin (filamentous actin), which is mainly found in the cellular cortex, stress fibers, and pseudopodia. It has been known that in almost all steps of cancer metastatic spread, the actin reorganization and reassembly are involved [1]. Since metastasis is the leading cause of cancer patient lethality, there are numerous efforts on developing treatment method to interfere with actin and its regulators in cancer research. For example, inhibition of the actin-bundling protein fascin effectively inhibits cancer metastasis in mice [2,3], indicating the effectiveness of targeting of actin regulation proteins for anticancer therapy.

In the last few decades, physical methods have been rapidly developing strategies for alternative cancer treatment because they have multiple advantages, including low toxicity and high temporal control. For example, tumor-treating field utilizes low-intensity medium-frequency alternating electric fields to interfere with the mitotic spindles in dividing cancer cells and has been approved by Food and Drug Administration to be used on glioblastoma [4–6]. Compared to electric field, magnetic field is less invasive and can provide better tissue penetration. It has been shown that even a weak magnetic field could affect new tissue formation in vivo [7] and a combination of static magnetic and electric field or a static magnetic field alone could both alleviate type 2 diabetes in mice [8,9]. Moreover, magnetic field has also been shown to be able to affect both purified actin and cellular actin [10–21]. For example, the 4-Hz oscillating magnetic fields could interrupt F-actin network in mesenchymal stem cells to inhibit their differentiation [21]. This is consistent with the well-known fact that F-actin is sensitive to mechanical stimuli by driving mechanical forces into biochemical signaling [22–25].

Here we chose 0.1-T and 0.4-T 250 r/m (~4.2 Hz) moderate intensity low-frequency rotating magnetic fields (LF-RMFs) to investigate their effects on breast cancer metastasis, the most common invasive cancer and the second leading cancer death in women. Combining multiple in vitro and in vivo experiments, comparing 2 breast cancer and 2 noncancer cell lines, as well as theoretical calculations, we found that LF-RMFs can directly but differentially affect F-actin in cancer vs. noncancer cells, which consequently decreases breast cancer cell attachment, migration, and invasion, as well as the reduced breast cancer metastasis in mice.

## Results

### LF-RMFs reduce F-actin in breast cancer cells

**Fig. 1.**
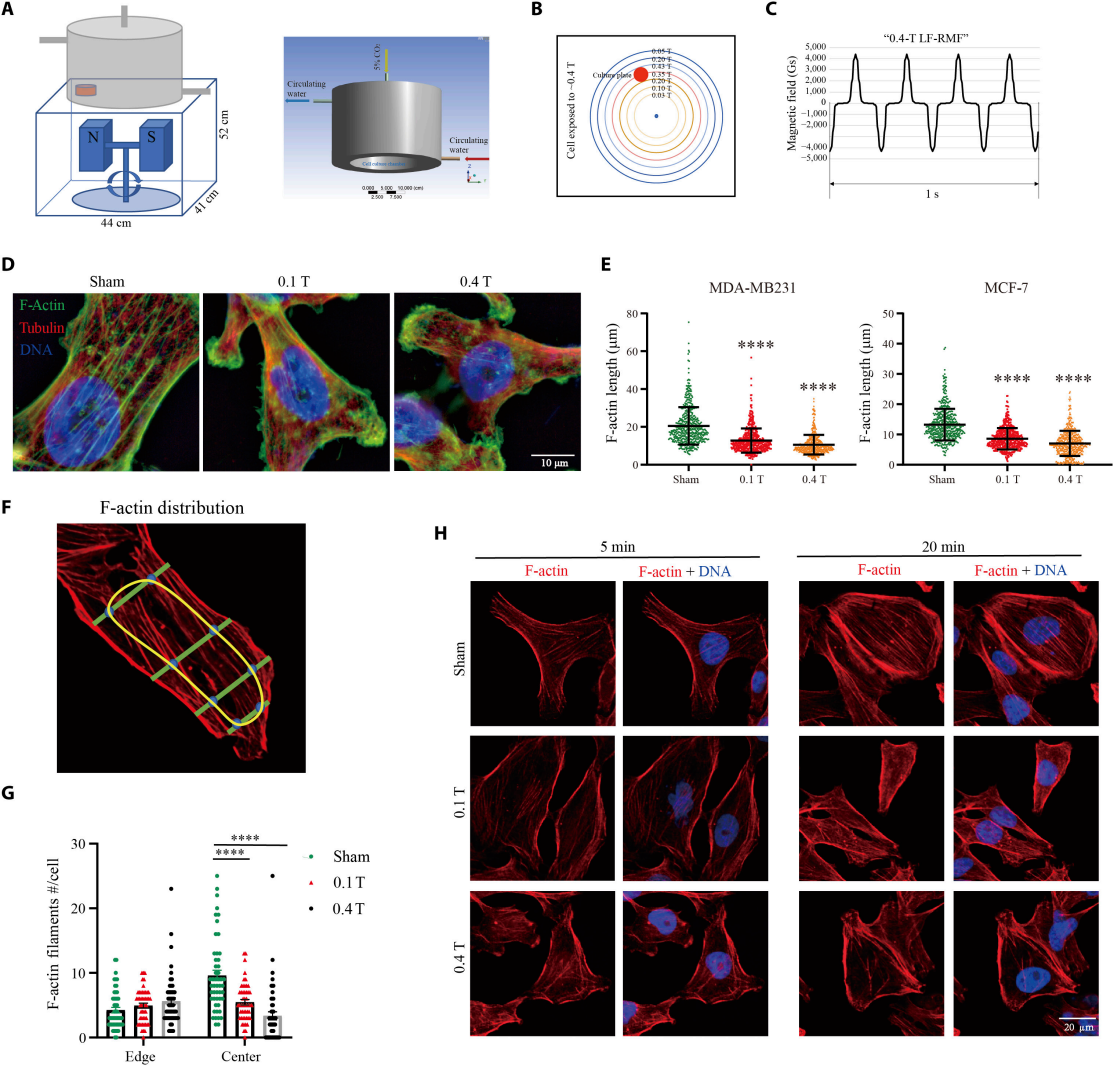
LF-RMFs reduce F-actin in breast cancer cells. (A) Illustration of experimental setup and design of the customized cell incubator. (B) Magnetic field intensity on the top of the “0.4-T LF-RMF” equipment. Red circle indicates where the cell culture dish was placed. (C) Magnetic field intensity changes at where the cells are. (D) Immunofluorescence images of MDA-MB231 cells after 6 h of sham or 0.1-T or 0.4-T 4.2-Hz RMF exposure. (E) Quantification of F-actin filament length. The length of 500 F-actin filaments was measured. Data are represented as means ± SD. (F) Areas were defined as center of the cells vs. surrounding areas. (G) Quantification of F-actin numbers in the center of the cells vs. surrounding areas. Data are represented as means ± SEM. (H) Immunofluorescence images of MDA-MB231 cells after sham or 0.1-T or 0.4-T 4.2-Hz RMF exposure for 5 or 20 min.****P < 0.0001. Scale bars: 20 μm.

To examine the effect of LF-RMFs on breast cancer cells, we custom-designed 3 sets of cell incubators to be used on 3 instruments: a sham control, a 0.1-T (Max) LF-RMF, and a 0.4 T (Max) LF-RMF (Fig. 1A to C and Fig. S1). Breast cancer MDA-MB231 and MCF-7 cells were plated to allow attachment before they were exposed to sham, 0.1-T LF-RMF, or 0.4 T LF-RMF. Immunofluorescence was performed after 6 h of exposure, which shows that the actin stress fibers were obviously disrupted by LF-RMFs, especially at 0.4 T (Fig. 1D and E and Fig. S2). Moreover, we quantified the F-actin filament number at the cell periphery vs. at the surrounding areas (Fig. 1F) and found that the F-actin in the cell center seems to be more obviously affected compared to the surrounding area (Fig. 1G). We also examined different time points at 5 and 20 min, and it is obvious that the F-actin in MDA-MB231 cells can be disrupted by LF-RMFs at as early as 5 min (Fig. 1H). In contrast, the microtubules in MDA-MB231 cells were not obviously affected by 0.4-T LF-RMF (Fig. S3).

### Time- and cell type-dependent effects of LF-RMFs on cellular F-actin

**Fig. 2.**
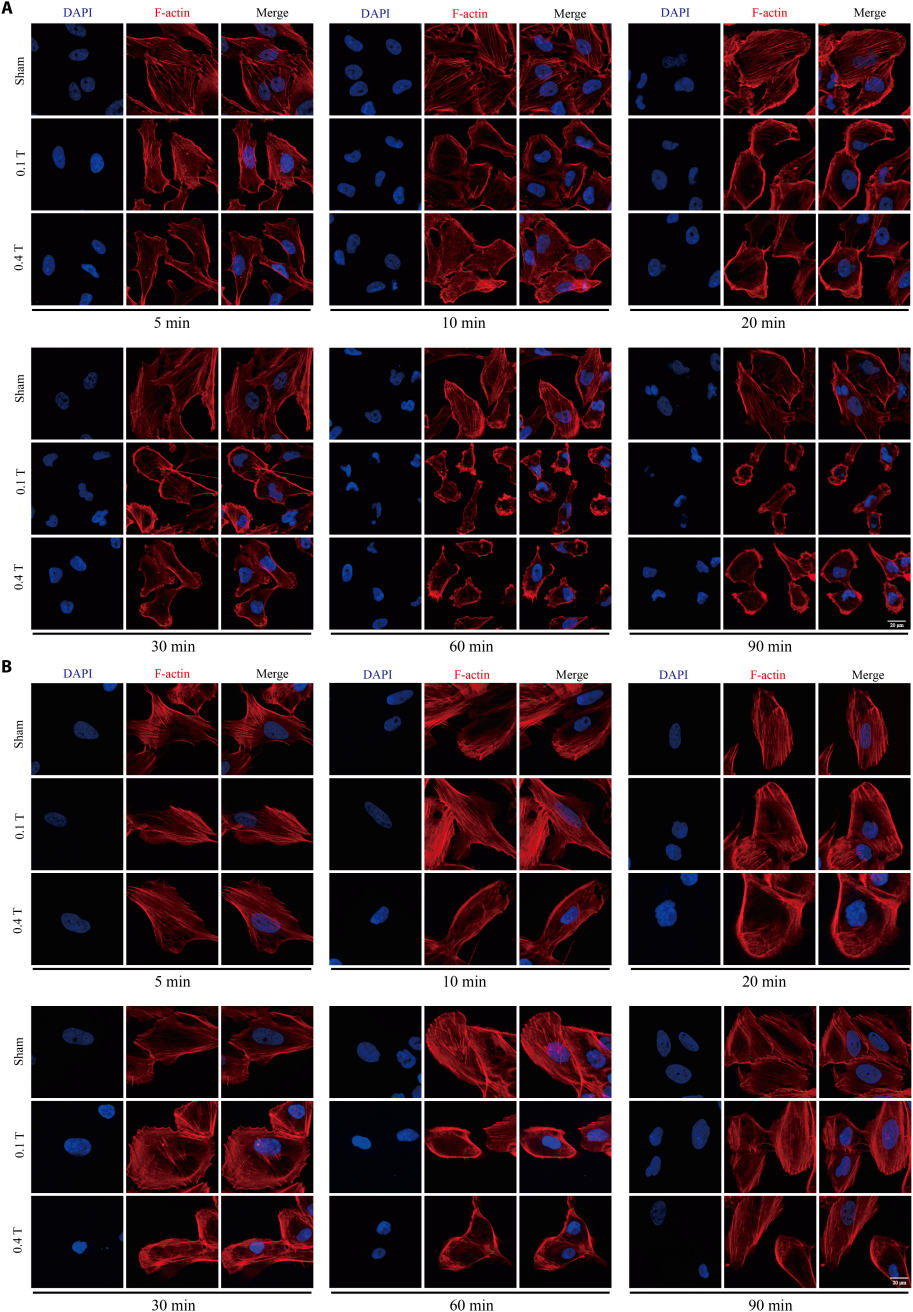
Time- and cell type-dependent effects of LF-RMFs on cellular F-actin. (A) MDA-MB231 breast cancer cells and (B) MCF10A breast noncancer cells were treated with sham, 0.1-T RMF, or 0.4-T RMF for different time points. Cells were fixed and stained with phalloidin and DAPI. Scale bar: 20 μm.

Since the actin distribution seemed different when the cells were treated with LF-RMFs for 6 h (Fig. 1D) vs. 5 or 20 min (Fig. 1H), we used a series of different time points to examine the effects of LF-RMFs on cellular actin. Using MDA-MB231 cells, it is obvious that the stress fibers were significantly reduced as early as 5 min (Fig. 2A), but there is an obvious switch from stress fibers to lamellipodia/ruffles from 10 to 60 min. At 90 min, the cells shrank and rounded up, similar to the effects of other actin drugs. We also tested 2 human noncancer cell lines, the normal breast MCF10A cells (Fig. 2B) and retinal pigment epithelial RPE1 cells (Fig. S4). The results show that the stress fibers in MCF10A and RPE1 cells are much more robust than MDA-MB231 because they are more resistant to LF-RMF perturbations. Moreover, they did not have the stress fibers to lamellipodia/ruffles switch or the roundup phenotypes at a later stage (Fig. 2B and Fig. S4) as did MDA-MB231 cells.

**Fig. 3.**
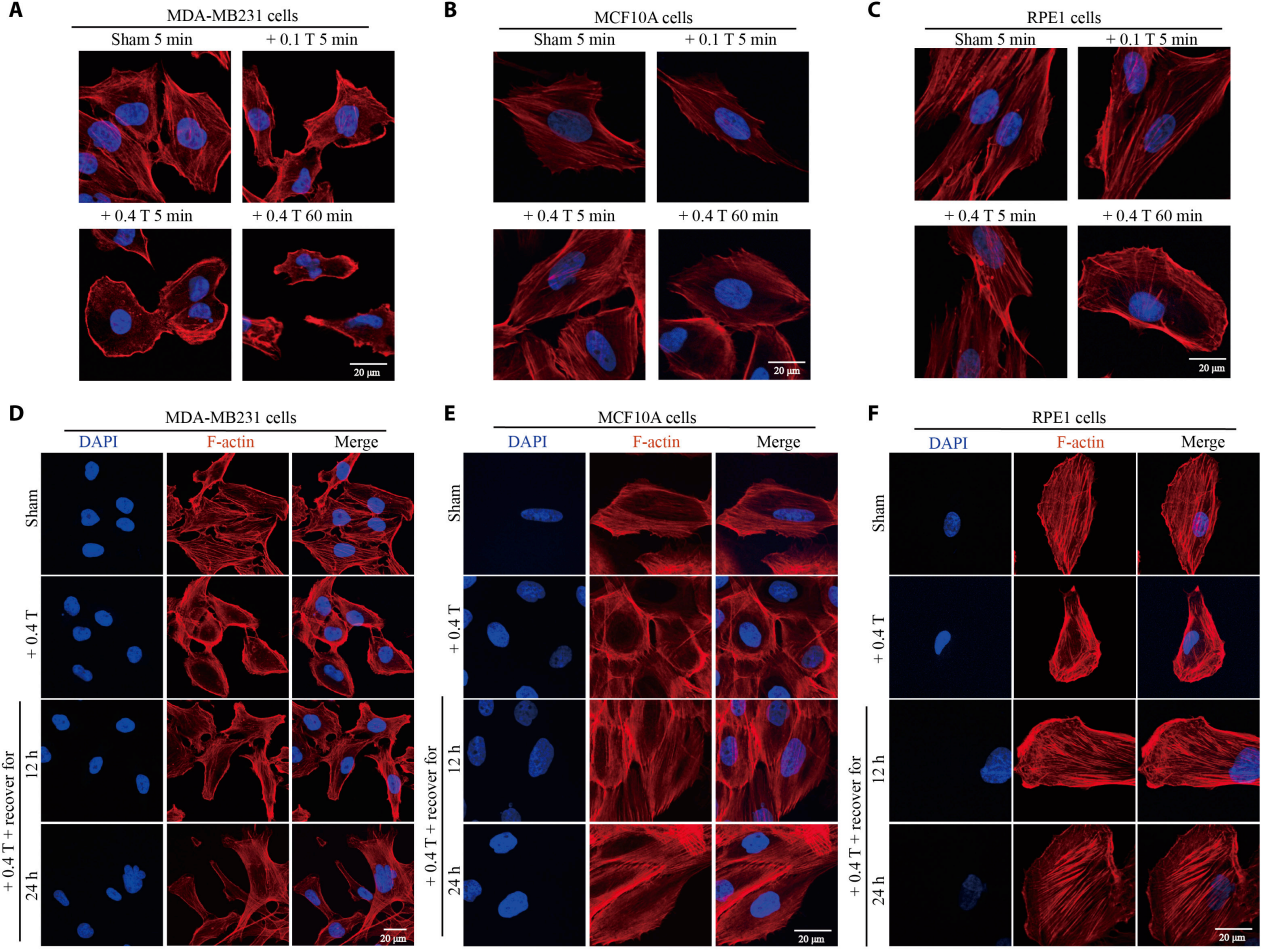
LF-RMFs differentially and reversibly affect F-actin in cancer vs. noncancerous cells. (A) MDA-MB231, (B) MCF10A, and (C) RPE1 cells were treated with 0.1- or 0.4-T LF-RMFs for 5 min or 0.4 T LF-RMF for 60 min. (D) MDA-MB231, (E) MCF10A, and (F) RPE1 cells were treated with 0.4-T LF-RMF for 1.5 h, with or without recovery for 12 or 24 h. Cells were stained with phalloidin and DAPI. Scale bar: 20 μm.

Next, we further side-by-side compared 3 different cell types—MDA-MB231 cells, MCF10A cells, and RPE1 cells—for different treatment time and/or recover time after magnetic field retrieval (Fig. 3). It is apparent that the F-actin changes were very obvious in the breast cancer MDA-MB231 cells, but not noncancer MCF10A cells or RPE1 cells (Fig. 3A to C and Fig. S5), confirming that the MDA-MB231 cells are much more sensitive to LF-RMFs compared with the 2 noncancerous cells. We also examined the recovery after 1.5 h of 0.4-T LF-RMF treatment. Our results show that the obvious cellular actin changes in MDA-MB231 caused by LF-RMF did not recover even after 24 h after LF-RMF retrieval, while the actin changes in MCF10A and RPE1 cells can soon recover (Fig. 3D to F and Fig. S6).

**Fig. 4.**
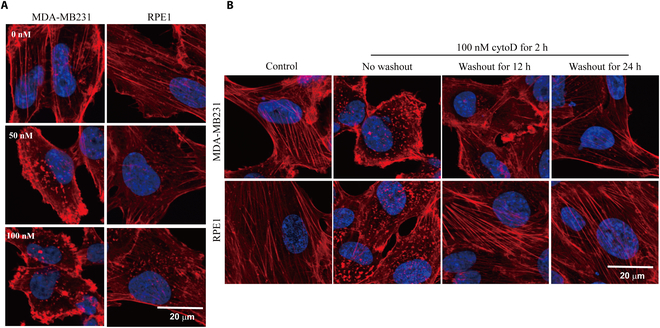
Cytochalasin D (cytoD) differentially and reversibly affects F-actin in cancer vs. noncancerous cells. (A) MDA-MB231 and RPE1 cells were treated with 0, 50, or 100 nM cytoD for 2 h before they were fixed and stained with phalloidin (red) and DAPI (blue). (B) MDA-MB231 and RPE1 cells were treated with 100 nM cytoD for 2 h, with or without additional washout to allow recovery for 12 or 24 h, before they were fixed and stained with phalloidin and DAPI. Scale bar: 20 μm.

To further examine the differential sensitivity of actin fibers in cancer vs. noncancer cells to perturbations, we used different concentrations of cytochalasin D (cytoD), as well as different recovery times after drug retrieval. It seems that cytoD induced a more significant effect on MDA-MB231 and MCF-7 breast cancer cells compared with the noncancer RPE1 cells (Fig. 4A and Fig. S7). Moreover, the RPE1 cells can fully recover after drug retrieval but not the MDA-MB231 and MCF-7 breast cancer cells (Fig. 4B and Fig. S8). These confirm that the F-actin in breast cancer cells is much more sensitive to drug or magnetic field perturbations compared with the noncancer cells.

### LF-RMFs significantly inhibit MDA-MB231 breast cancer cell migration, invasion, attachment, and spreading in vitro

**Fig. 5.**
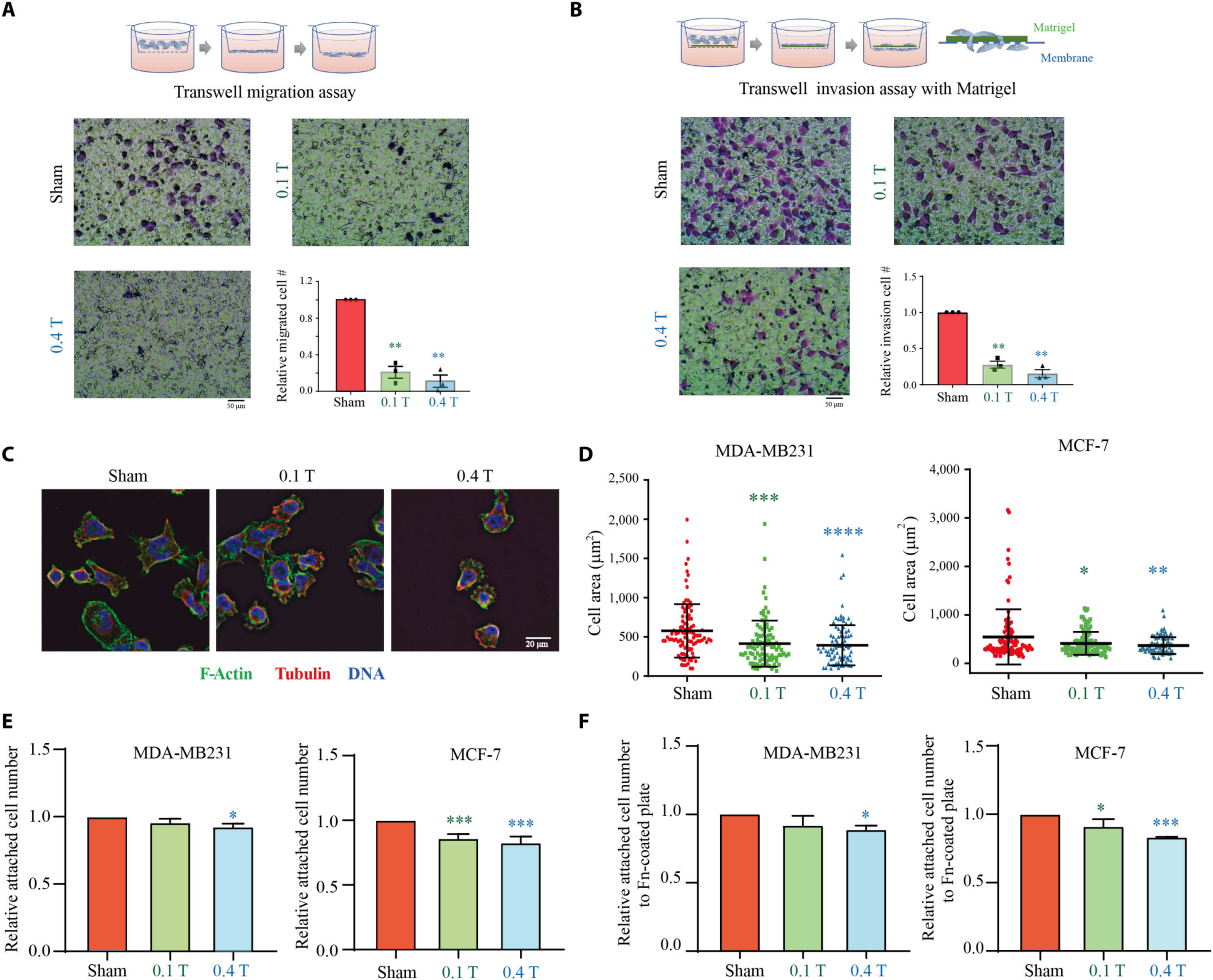
LF-RMFs significantly inhibit MDA-MB231 breast cancer cell migration, invasion, attachment and spreading in vitro. (A) Experimental illustration, representative images, and quantification of relative numbers of migrated cells in Transwell migration assays in vitro. Data are represented as means ± SEM. (B) Transwell invasion assays with Matrigel. Data are represented as means ± SEM. (C) Immunofluorescent images of MDA-MB231 cells stained with phalloidin (green), anti-tubulin antibody (red), and DAPI (blue) after 6 h of RMF treatment. Scale bar: 20 μm. (D) Quantification of cell area assessed through phalloidin staining in MDA-MB231 cells after 6 h of sham or RMF treatment. Both MDA-MB231 and MCF-7 cells were quantified. Data are represented as means ± SD. (E) Quantification of relative attached cell number after 18 h of sham or RMF treatment. Data are represented as means ± SD. (F) Quantification of cell attachment experiments using fibronectin (Fn)-coated plates show that LF-RMFs reduced both MDA-MB231 and MCF-7 breast cancer cell attachment after 18 h of treatment. Data are represented as means ± SD. Experiments have been repeated 3 times. *P < 0.05, **P < 0.01, ***P < 0.001, and ****P <0.0001.

Since F-actin is essential for cell migration and invasion, we did 3 in vitro experiments to test whether LF-RMFs can affect MDA-MB231 breast cancer cell migration and invasion. We used the wound healing migration assay (Fig. S9), Transwell migration assay (Fig. 5A) and Transwell invasion assay with Matrigel (Fig. 5B), a mixture of laminin and collagen, to provide a 3-dimensional cell culture condition. It is obvious that both 0.1-T and 0.4-T LF-RMFs have significant inhibition effects on MDA-MB231 cell migration and invasion (Fig. 5A and B and Fig. S9).

Next, we examined the effects of LF-RMF on breast cancer cell attachment and spreading, in which F-actin also plays a central role. We exposed MDA-MB231 and MCF-7 breast cancer cells to LF-RMFs right after they were plated on coverslips in the tissue culture plates. After 6 h, the cells were fixed and stained. We observed that both MDA-MB231 and MCF-7 (Figs. 5C and S2) had decreased attachment and spreading after LF-RMF treatment, especially the 0.4 T. Quantification of the cell area shows that both 0.1-T and 0.4 T LF-RMFs reduced the cell area (Fig. 5D). In addition, we tested the relative attached cell numbers after 18 h of LF-RMF treatment and found that the attached cell numbers were also reduced (Fig. 5E). Moreover, to mimic the in vivo cell attachment condition, we tested cell attachment on surfaces coated with one of the extracellular matrix proteins, fibronectin (Fn). We found that the cell attachment on Fn-coated surface was also reduced in both types of breast cancer cell lines by LF-RMFs (Fig. 5F). Therefore, our results indicate that MDA-MB231 and MCF-7 cell attachment can be reduced by 0.1-T and 0.4-T LF-RMFs, especially the 0.4-T LF-RMF.

### LF-RMFs inhibit breast cancer metastasis, prolong survival time, and improve physical conditions of mice bearing MDA-MB231 breast cancer cells

Since our cellular assays show that multiple processes involved in cancer metastasis are inhibited by LF-RMFs, including cell migration, invasion, attachment, and spreading, we next investigated whether LF-RMFs can affect breast cancer metastasis in vivo. The LF-RMF acts on both cancer cells and normal cells. Although the normal cells are much less sensitive to this perturbation, they are also affected to some extent. However, the normal cells have a much better recovery ability after magnetic field retrieval compared with the cancer cells (Fig. 3D to F and Fig. S6). We hypothesize that using intermittent treatment, we can achieve a maximum perturbation on the cancer cells and a minimal effect on the normal cells.

**Fig. 6.**
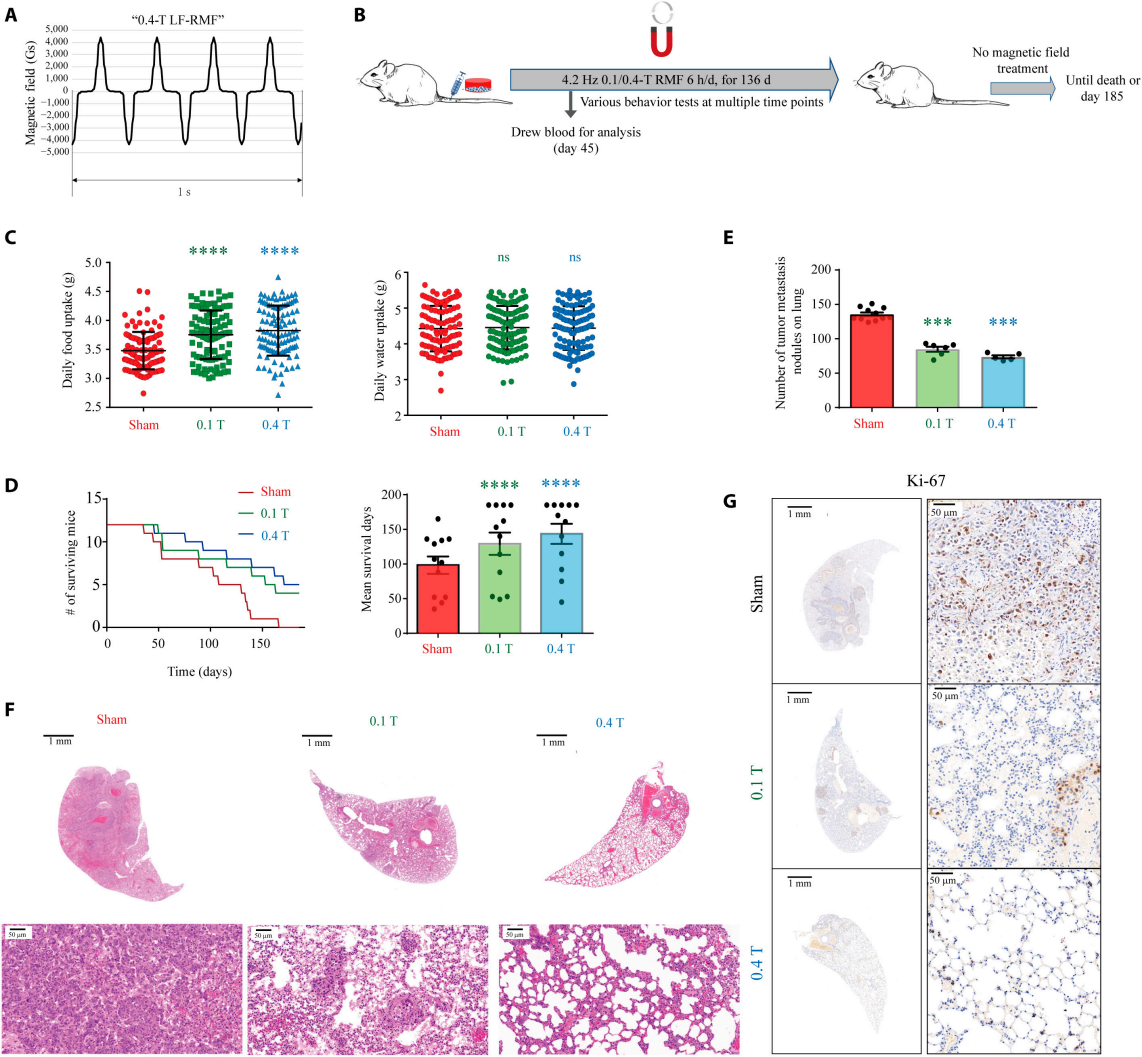
LF-RMFs significantly inhibit MDA-MB231 breast cancer metastasis and prolonged the mice survival time. (A) Magnetic field induction as a function of time. (B) Experimental procedure. MDA-MB231 cells were injected intravenously through the tail vein. (C) Average daily food and water intake of mice in different groups. Data are represented as means ± SD. (D) LF-RMFs significantly prolonged survival time of mice bearing MDA-MB231 breast cancer cells. Data are represented as means ± SEM. (E) LF-RMFs significantly reduced number of tumor metastasis nodules on the lung tissues. The lung tissues collected before day 140 were analyzed for metastasis module numbers. For sham control, *n* = 11; for 0.1 T, *n* = 6; for 0.4 T, *n* = 5. Data are represented as means ± SEM. (F) Representative lung section HE staining shows that LF-RMF-treated mice have relative normal lung tissues. (G) Representative lung section Ki-67 staining shows that LF-RMF-treated mice have much reduced proliferating cancer modules in their lung tissues. ***P < 0.001, ****P < 0.0001; ns, not significant.

We used female BALB/c (nu/nu) nude mice and injected 5 × 10^6^ MDA-MB231 cells into their tail veins to develop breast cancer cell metastasis mice model. The mice were then treated with sham, 0.1-T LF-RMF, or 0.4-T LF-RMF for 6 h/d, for 136 d (Fig. 6A and B) (*n* = 12 for each group). Their body weight (Fig. S10) and water and food consumption (Fig. 6C) were measured every day, and their blood samples were collected for blood routine test on the 45th day after intravenous injection (Fig. S11). No statistical abnormality in the blood was revealed, but we observed obvious improvement of their physical conditions after LF-RMF treatment. To get a more accurate evaluation, we performed multiple behavior tests, including the balance beam test, grip test, and open-field test, which show that LF-RMFs could improve the motor coordination, muscular strength, and exploratory activity of MDA-MB231–bearing mice (Fig. S12). These results and the increased food consumption of LF-RMF-treated mice indicate that LF-RMFs could improve the overall health conditions of these breast cancer cell metastatic mice.

After 136 d of LF-RMF treatment, the mice were fed normally, without more LF-RMF treatment, until all mice in the sham control group died before day 165 (Fig. 6D). However, there were still 4 and 6 mice alive in the 0.1-T and 0.4-T RMF-treated groups at that time, respectively. We continued to feed them and monitored their growth until day 185 before the remaining mice were sacrificed. Statistical results show that the 0.1-T LF-RMF has prolonged the mice survival time by 31.5% (average survival time of 0.1-T LF-RMF-treated mice is 129.33 d vs. 98.33 d for the sham control; *n* = 12; *P* < 0.0001 by Student *t* test) and the 0.4-T LF-RMF has prolonged the mice survival time by 46.0% (average survival time of 0.4-T LF-RMF-treated mice is 143.58 d vs. 98.33 d for the sham control; *n* = 12; *P* < 0.0001 by Student *t* test) (Fig. 6D).

Since MDA-MB231 breast cancer cells were injected into these mice to induce metastasis, we examined the mice tissues to see whether LF-RMFs affect their metastatic condition. It is obvious that the number of metastatic nodules on the lungs of these mice was significantly reduced by LF-RMFs (Fig. 6E). We further examined the lung tissues with HE (hematoxylin and eosin) (Fig. 6F), and Ki-67 staining (Fig. 6G), a commonly used marker for cell proliferation in cancer. Our HE staining results show that both 0.1-T and 0.4-T LF-RMFs could reduce the lung metastasis, while the 0.4-T LF-RMF has a more significant effect than the 0.1-T one (Fig. 6F). The lung tissues in the 0.4-T-treated mice had remained their normal appearance. Moreover, 0.4 T has a more significant effect than 0.1 T in reducing the Ki-67 staining in lung tissues (Fig. 6G). We have also examined 3 additional cancer markers, the proliferating cell nuclear antigen, epidermal growth factor receptor, and Vimentin, which all confirmed that the LF-RMFs could inhibit the breast cancer metastasis in lung tissues, and the 0.4-T LF-RMF had a more significant effect than the 0.1-T LF-RMF (Fig. S13).

### RHO activity and cell cycle progression were disrupted by LF-RMFs in breast cancer cells

**Fig. 7.**
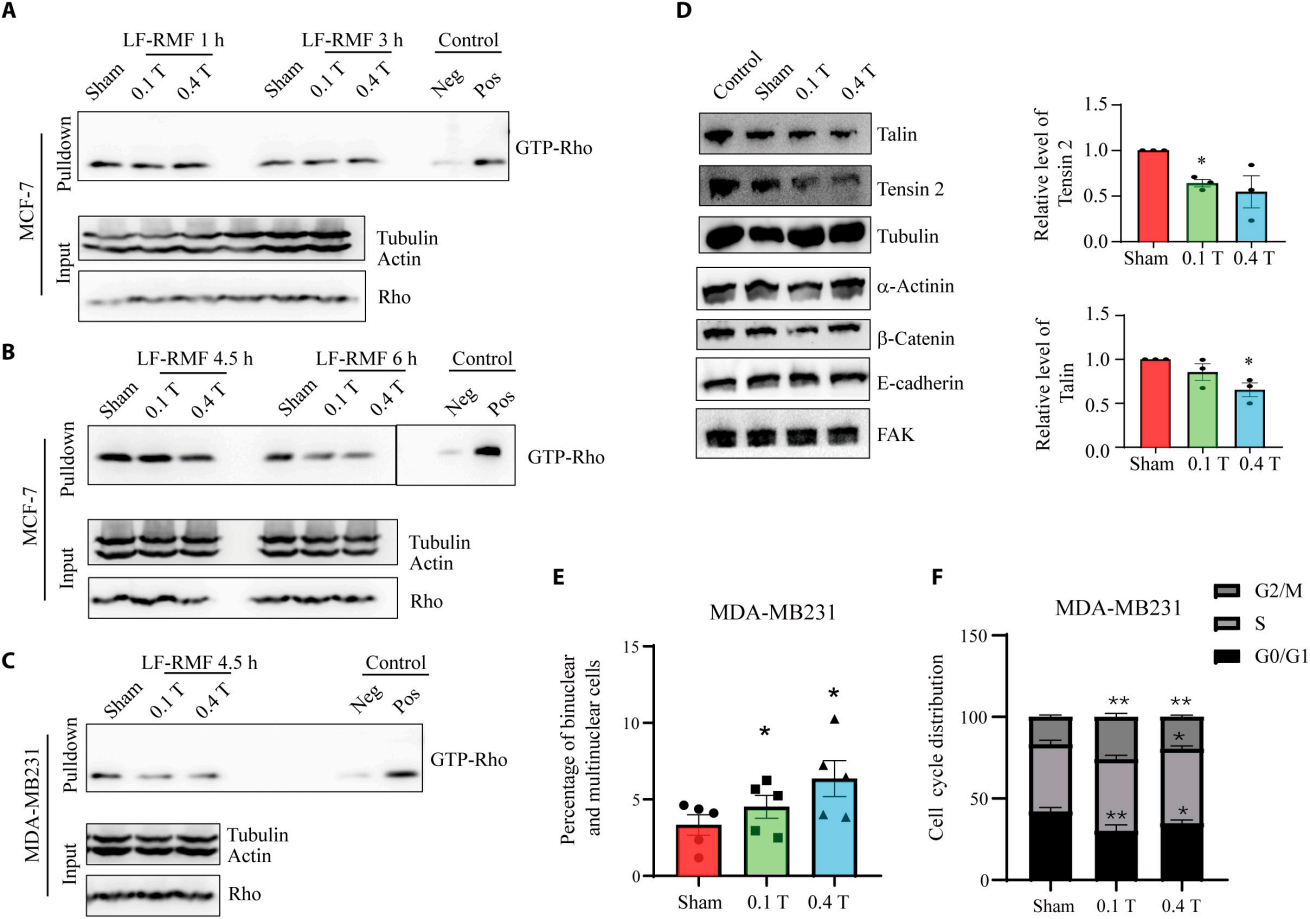
RHO activity and cell cycle progression were disrupted by LF-RMFs in breast cancer cells. (A to C) RHO activation assay shows that RMF could reduce RHO activation in MCF7 and MDA-MB231 cells after 4.5 h. Neg, negative control. Pos, positive control. (D) Representative Western blots and the quantification show that Talin and Tensin 2 levels are reduced by LF-RMF treatment in MCF-7 cells. Data are represented as means ± SEM. *n* = 3. (E) Quantification of the percentage of binucleated and multinucleated MDA-MB231 cells. Data are represented as means ± SEM. (F) The cell cycle distribution after being treated with sham, 0.1-T LF-RMF, or 0.4-T LF-RMF for 30 h. Data are represented as means ± SD. *P < 0.05 and **P < 0.01.

It has been reported by Wosik et al. [20] that the magnetic force produced by medium intensity gradient static magnetic field could cause the rearrangement of actin cytoskeleton of macrophages and change cell morphology, which was similar to Rho inhibition. Since Rho guanosine triphosphatases (GTPases) play important roles in cell adhesion, migration, and invasion [26–28], we next examined whether LF-RMF could affect Rho guanosine triphosphatase activity in breast cancer cells. We first performed Rho activation assays after treating MCF-7 cells for 1 or 3 h but did not observe obvious changes (Fig. 7A). However, when we prolonged the treatment time to 4.5 h, the Rho activity was reduced, which is even more obvious at 6 h (Fig. 7B). We also treated MDA-MB231 with LF-RMF for 4.5 h and observed reduced Rho activity as well (Fig. 7C). In addition, we examined some cell attachment- and invasion-related markers and found that the focal adhesion structural protein Talin and Tensin 2 levels were both decreased by LF-RMF treatment (Fig. 7D). Moreover, not surprisingly, since LF-RMFs affect actin and related signaling pathways, they also increase the binucleated cells (Fig. 7E) and arrest cell cycle progression in MDA-MB231 cells (Fig. 7F).

### LF-RMFs directly affect F-actin polymerization and depolymerization

**Fig. 8.**
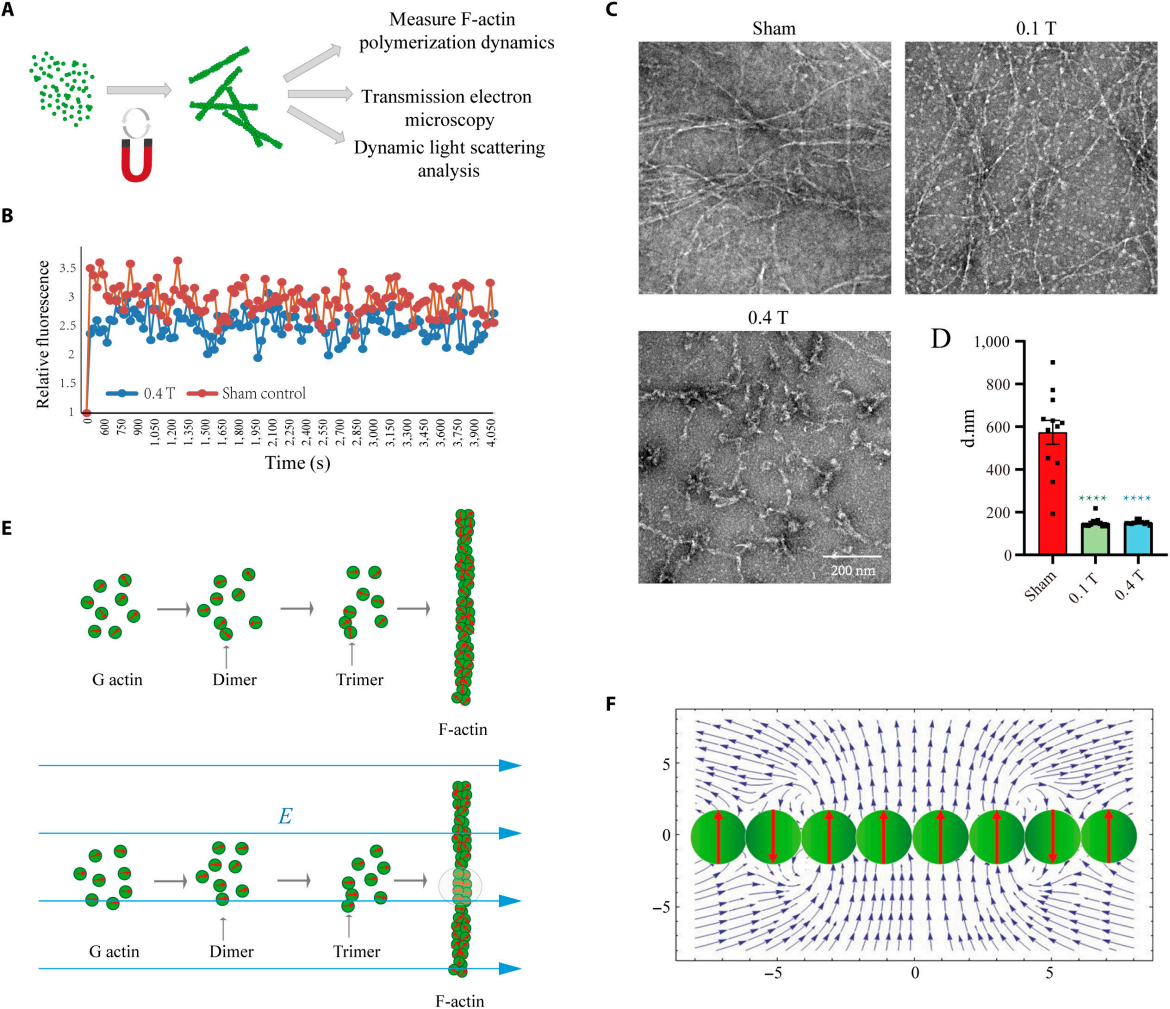
LF-RMFs directly affect actin polymerization in vitro*.* (A) Experimental procedures illustration. (B) Pyrene-actin assay shows that the 0.4-T LF-RMF treatment for 5 min can directly inhibit actin polymerization in vitro*.* (C) Representative TEM images of F-actin assembled in sham, 0.1-T LF-RMF, or 0.4-T LF-RMF. Scale bar: 200 nm. (D) DLS shows the particle size distribution of actin after 10 min of assembly in sham, 0.1-T LF-RMF, or 0.4-T LF-RMF. Data are represented as means ± SEM. ****P < 0.0001. (E) Actin polymerization with or without electric field. The red arrows represent the electric dipole moments of G-actin. Blue arrows are the electric field lines. Shadow area is an F-actin domain with parallel orientation of the dipole moments. In this area, the G-actin binding energy is reduced by the dipole-dipole interaction energy. (F) Sketch of a fraction of F-actin containing a domain with parallel orientation of dipole moments of G-actin monomers (4 central green circles). Calculated vector field, *E*, of 8 electric dipoles (↑↓↑↑↑↑↓↑) is shown by the blue arrows. In the domain vicinity, the energy of the stray electric field weakens the G-actin binding energy in the chain. On the horizontal scale, unity corresponds to the length of 1 G-actin monomer, 2.7 nm.

Since LF-RMFs can affect cellular F-actin in as short as 5 min (Figs. 1 to 3), while the RHO activity needs a much longer time (Fig. 7A to C), we hypothesize that actin may be the direct target of LF-RMFs. Next, we performed in vitro actin polymerization assays using pyrene-actin to measure its dynamics and used transmission electron microscopy (TEM) to observe their assembled structures (Fig. 8A to C). Because of the technical limitations, we cannot monitor this polymerization process while the reactions were exposed to LF-RMF, so we initiated the actin polymerization reactions in the presence of sham, 0.1-T LF-RMF, or 0.4-T LF-RMF for 5 min and then used a fluorescent spectrophotometer to monitor the formation process of F-actin fibers, which was reflected by the fluorescence level of pyrene-actin. Our results show that even the short treatment of actin polymerization reaction by 0.4-T LF-RMF for only 5 min was enough to reduce the level of F-actin polymerization (Fig. 8B), which is consistent with our previously observed cellular F-actin changes in cells (Figs. 1 to 3).

To confirm the effects of RMFs on actin polymerization, we used TEM and dynamic light scattering (DLS) to examine the actin polymerization in the absence or presence of RMFs for 10 min. Both experiments show that F-actin formation is inhibited by RMF treatment (Fig. 8C and D). In the TEM experiment, the actin filaments are abundant and robust in the sham group, which were greatly reduced by RMF treatment (Fig. 8C). In the DLS experiment, the total particle size in the sham group has the highest average size (573.77 nm), which was significantly reduced by RMF treatment (Fig. 8D).

We hypothesize that RMF-induced electric field may cause these effects. When a cell dish is placed in the RMF, an electromotive force *V* rises, which reads as

$$V=-\frac{d(\text{BS})}{\text{dt}}=-\pi \text{Rr}\omega B$$ (1)

where *B* is the magnetic induction of the RMF, *R =* 20 cm is the rotation radius, *r* = 9 mm is the cell dish radius, and ω = 2πν is the angular velocity of rotation. The derivative of the magnetic flux with respect to time refers to how the magnetic field changes with the time. The cells we used are attached to the bottom of the tissue culture plate so that they can be considered as flat 2-dimensional disks. By definition $V=\oint \vec{E}\vec{\text{dl}}=2\pi \text{rE}$, from which, considering Eq. 1, the induced electric field amplitude, *E* reads as

E=πνBR (2)

Estimation of Eq. 2 for ν = 4.2 Hz, *R* = 20 cm, and *B* = 0.4 T gives *E* = 1.1 V/m. It is known that the building block G actin polymerizes into semiflexible F-actin filaments (Fig. 8E). When actin polymerizes in the presence of electric field, there is a torque on the actin dipole moment, $\vec{M}=\left[\vec{p}\vec{E}\right]$, which tends to align the G-actins with the field and can lead to the appearance of domains with the parallel orientation of the dipole moments of G-actin (Fig. 8E).

To analyze the role of a domain with the parallel-oriented dipole moments in F-actin stability, we calculated the distribution (vector field) of the electric field of the dipole chain consisting of 8 G-actin monomers oriented as shown on Fig. 8F. Here, the domain of 4 G-actin monomers, with parallel orientation of the dipole moments, generates a local stray electric field, E (the blue quasi-parallel field lines) with a positive energy that is proportional to E^2^, thereby weakening the negative binding G-G actin energy. The calculations were performed with the Wolfram Mathematica software.

To evaluate the contribution of the domain formation in F-actin, we can consider 2 parallel dipoles with moment *p* = 75 Debye = 75⋅3.336 × 10^−30^ C m = 2.5 × 10^−28^ C m, separated by the distance *l* = 2.7 nm (monomer length). Their interaction energy is

$$W_{\text{dip}}=\frac{p^{2}}{4{\pi \varepsilon }_{0}l^{3}}$$ (3)

where *ε*_0_ is the permittivity of free space. Estimations of Eq. 3 give *W_dip_* = 2.85 ⋅10^−20^ J, which is larger than the thermal fluctuation energy, *k_B_T* (where *k_B_* is the Boltzmann constant and *T* is the temperature): *W_dip_*/ *k_B_T* = 6.9. Thus, these dipoles (G-actin monomers) repulse each other with the energy 17.16 kJ/mol (=*W*_dip_ *N_A_*, where *N_A_* is the Avogadro’s number). The GG actin repulsion force can be estimated as *F_GG_ ≈ W_dip_/l* = 10 pN. Importantly, a force of the same order of value (15 to 27 pN) can significantly change both the bond lifetimes of G-actin–G-actin (GG) and G-actin–F-actin (GF) interactions [29].

For the effect of the GG dipole actin interaction on the binding energy, since the binding energy of G-actin monomers is *W* = −27.81 kJ/mol [30], the effective binding energy is reduced by G-actin dipole-dipole repulsion and reads as

$$W_{\text{ef}}=W+\frac{p^{2}N_{A}}{4{\pi \varepsilon }_{0}l^{3}}\approx -\;10.21\;\mathrm{kJ}/\mathrm{mol}$$ (4)

This implies that the ratio of the effective binding energy to the thermal fluctuation energy is *W_ef_*/*k_B_T* = 4.1. Therefore, the bond survival probability is decreased by the GG dipole interaction. Thus, the estimated contribution of the dipole-dipole interaction to the binding energy of actin (Eq. 4) allows us to conclude that in the presence of an electric field induced by the RMF, F-actin becomes less stable. In other words, in the presence of the RMF, an F-actin assembly operates just above the thermal fluctuation energy limit, *W_ef_*/*k_B_T* = 4.1, and a small mechanical energy input or a force impulse will disturb the stability of F-actin filaments by breaking it into parts.

## Discussion

**Fig. 9.**
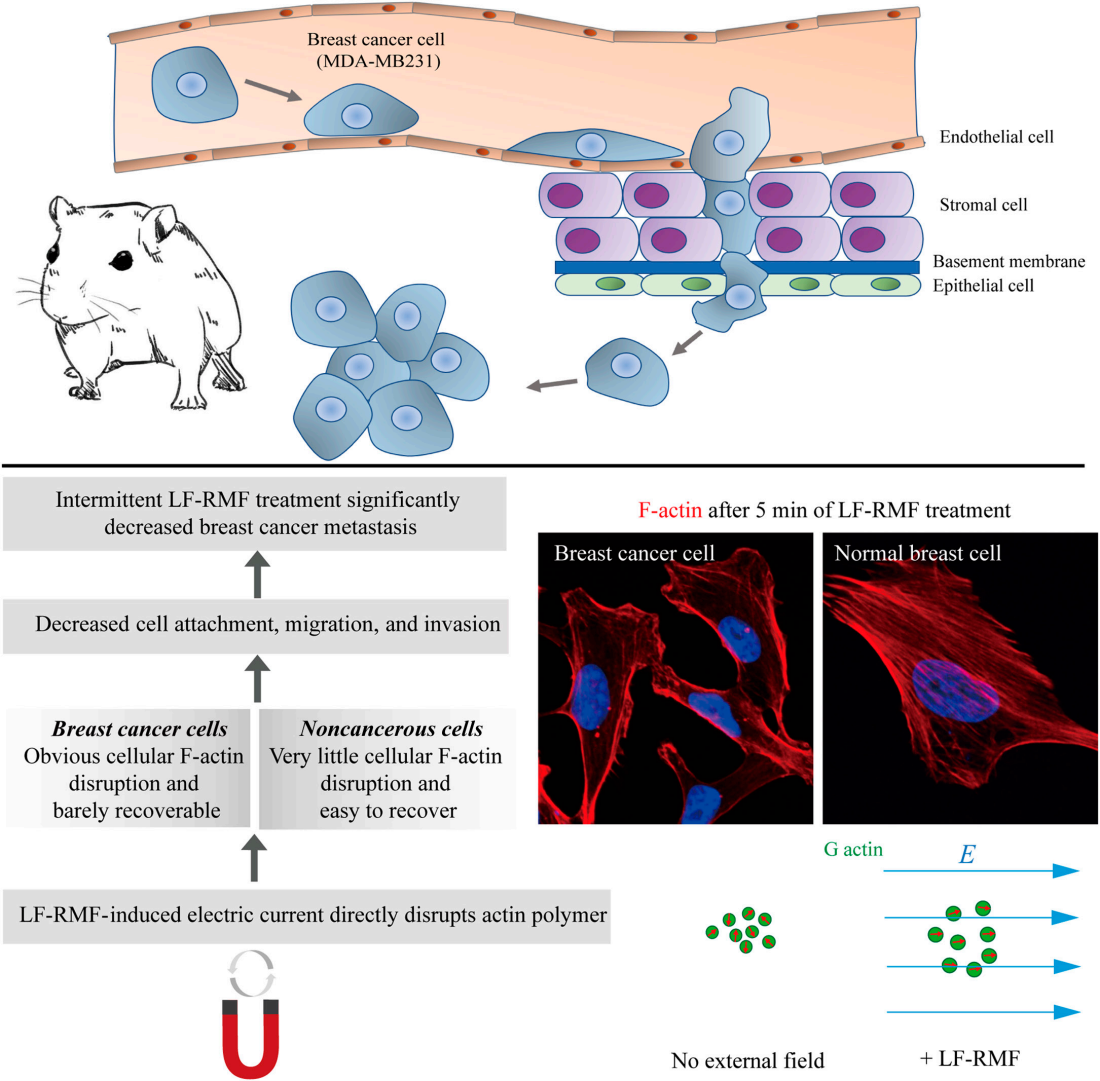
A model illustrates that LF-RMFs decrease F-actin to inhibit breast cancer cell attachment, migration, and invasion, which reduces breast cancer metastasis in vivo.

The actin dipole moments are randomly oriented when there is no electric field, while an electric field tends to align them parallelly. Here, we show that 0.1- and 0.4-T, 4.2-Hz LF-RMF-induced electric field could directly decrease F-actin stability. The estimated change of the actin binding energy provoked by the LF-RMF is sufficient to destabilize F-actin polymerization process. This destabilization leads to F-actin shortening and polymerization dynamics changes, which consequently reduces the ability of cells to adhere to a surface and spread. In vivo, these effects manifest themselves in reducing breast cancer metastasis and increasing the survival rate of tumor-bearing mice (Fig. 9).

It is interesting that we found that the cellular actin filaments in breast cancer cells are much more sensitive and respond to LF-RMFs very differently from noncancerous cells. First of all, the MDA-MB231 cancer cells are much more sensitive to LF-RMFs, whose stress fibers are obviously reduced as early as 5 min after LF-RMF treatment. Secondly, the MDA-MB231 cancer cells have time-dependent stress fiber to lamellipodia switch, while the 2 noncancerous cells do not. This is consistent with the metastatic characteristic of the MDA-MB231 cancer cells. Thirdly, the actin abnormalities and cell shape changes in the MDA-MB231 cancer cells are not recoverable at 24 h after treatment, while the subtle changes in the 2 noncancerous cells can fully recover. Therefore, when used intermittently, LF-RMF could interrupt F-actin in breast cancer cells while having minimum effects on noncancerous cells. Compared to current actin drugs, many of which have obvious cell toxicity, the high-temporal control, low-toxicity, and high-penetration characteristics of magnetic fields make them a superior research tool to investigate actin dynamics in cells and animals and inhibit breast cancer metastasis and maybe other types of cancer as well.

Because of the intrinsic diamagnetic anisotropy of peptide bond [31], the diamagnetic anisotropy of filamentous cytoskeletons can be additive so that they are generally susceptible to external magnetic fields, both in vitro [32,33] and in cells [34–39]. Multiple studies have shown that magnetic fields can target the microtubules and mitotic spindle [37,38,40–45]. F-actin is known to be sensitive to mechanical stimuli and plays essential roles in multiple physiological and pathological processes, including cancer metastasis. High-magnetic field-induced F-actin changes have been shown to affect cell behavior [46]. On the basis of theoretical and experimental evidences [21,47], the 4-Hz oscillating magnetic fields could interrupt F-actin network in mesenchymal stem cells to inhibit their differentiation [21], which is consistent with a previous study showing that the relaxation time of F-actin network in cells is about 0.1 to 1 s [47]. Therefore, we think F-actin network may have resonance with external magnetic field of 1- to 10-Hz frequency. In fact, it has been shown in *Dictyostelium* cells that there is an intrinsic oscillatory process in the cellular actin system, which has a clear resonance at a stimulation period of 20 s in that specific cell type [48]. Although we only tested the frequency of 4.2 Hz (max rotation speed) in this study because of the limitation of our equipment, we hypothesize that the F-actin network of the studied cells may have resonance with external LF-RMF of 1- to 10-Hz frequencies. In fact, some LF-RMF studies using a faster rotation speed of 7.5 Hz, also at 0.4 T, have shown to have anticancer potentials in mice bearing lung cancer, melanoma, and hepatocellular carcinoma [49–51]. We hypothesize that LF-RMF-induced F-actin disruption is likely to be one of the fundamental mechanisms for these cancer metastasis inhibitions. Although we did not observe obvious microtubule cytoskeleton in this study, the effects of LF-RMFs of different intensities and frequencies should also be systematically addressed in the future. It is possible that microtubules may be responsive to magnetic field parameters that are different from the actin cytoskeleton.

It should be mentioned that although magnetic fields have superior tissue penetration, it also means that they do not have a precisely control-focused area. However, since the majority of our bodies are composed of very weak diamagnetic materials, including water, lipids, and proteins, which are not sensitive to weak to moderate magnetic fields. Therefore, weak to moderate magnetic fields do not have obvious perturbations on them. More importantly, we revealed that the F-actin in noncancerous breast cells is much less sensitive than that in breast cancer cells, which indicate that the normal cells in our human bodies are less likely to be agitated by these magnetic fields.

In summary, our study shows that 0.1/0.4-T, 4.2-Hz RMFs could interrupt actin filament to inhibit breast cancer cell attachment and migration in vitro and reduce breast cancer metastasis in vivo. The survival rate and physical conditions of breast cancer tumor-bearing mice were also significantly improved by RMF treatment. This intermittent LF-RMF treatment (a few hours per day) has the potential to be developed into a promising cancer treatment method, which could effectively inhibit cancer metastasis while having a minimum effect on normal cells. The advantage of its high penetration through tissues, the easy removal of magnetic field at any time, as well as its local applications to specific part of the bodies make it a very unique and powerful physical tool that can afford high temporal and spatial control over a large variety of cancer types. Therefore, people are encouraged to investigate more cancer cell types, magnetic field frequencies, as well as increased magnetic field intensities to further explore the future clinical potentials of these low-frequency RMFs, which could likely lead to significantly improved therapeutic outcomes.

## Materials and Methods

### Cell culture

The cell lines MDA-MB231 (RRID: CVCL_0062) and MCF-7 (RRID: CVCL_0031) were from American Type Culture Collection. Cell line identity was confirmed and cultured in Dulbecco’s modified Eagle medium (DMEM) supplemented with 10% fetal bovine serum (CLARK Bioscience, FB25015).

### Cell synchronization assay

MDA-MB231 cells were plated on coverslips or in 35-mm cell culture dishes at 1 × 10^5^ cells/ml for 24 h. Single thymidine was used for synchronization. Thymidine (2.5 mM; Sigma-Aldrich, T1895) was added to cells for 17 h before washed with phosphate-buffered saline (PBS) to remove thymidine. After adding fresh DMEM, the cells were treated with RMF or sham immediately for another 30 h. The treated cells were collected for cell cycle detection or immunofluorescence analysis.

### Cell cycle analysis

MDA-MB231 cells blocked by single thymidine were released in fresh DMEM and immediately exposed to RMF or sham for 30 h. Cells were collected and trypsinized with 0.25% trypsin/EDTA and washed with ice-cold PBS 3 times before they were fixed with −20 ^o^C 70% ethanol overnight. After fixation, the cells were washed with PBS to remove ethanol, and stained with propidium iodide solution (BD Pharmingen, San Diego, CA, USA). The data were acquired by flow cytometry (CytoFLEX, Beckman Coulter, Brea, CA, USA) and analyzed by MODFIT LT (Verity Software House, LA, CA, USA, RRID: SCR_016106).

### Immunofluorescence

Cells plated on coverslips were washed with PBS and fixed in 4% formaldehyde at room temperature for 20 min. After block by AbDil-Tx (TBS [0.1% Triton X-100, 2% bovin serum albumin, and 0.05% sodium azide]) for 30 min at room temperature, cells were subjected to immunofluorescence using β-tubulin antibody (TransGen Biotech, HC101-02) and Alexa Fluor 488-conjugated anti-mouse IgG (Invitrogen, A-21202). F-actin was stained with Alexa Fluor™ 594 phalloidin (Invitrogen, A12381) for 0.5 h at room temperature. Cells were then stained with 300 nM 4′,6-diamidino-2-phenylindole (DAPI) at room temperature for 2 min before mounted with Pro-Long Gold Antifade (Invitrogen, P36930). Images were taken using an Olympus (SpinSR10, Olympus, Tokyo, Japan) fluorescence microscope. Percentage of binuclear and multinuclear cells was quantified on the basis of the immunofluorescence images.

### Cell culture wound closure assay

Cells were plated at 3× 10^5^ cells/ml in a 3.5-cm cell culture dish and grew to 100% confluence in a 37 °C, 5% CO_2_ incubator for 24 h. Then, a vertical wound scratch throughout the middle of cell monolayer was made using a 200-μl pipette tip. The media and cell debris were carefully aspirated before replacing with culture media without fetal calf serum (FCS). An initial picture was taken immediately by an inverted microscope before the cell culture dishes were exposed to sham, 0.1-T RMF, or 0.4-T RMF for 24 h. Pictures were taken every 6 h to record the process of wound closure.

### Cell migration and invasion assay

Migration of MDA-MB231cells were assessed by using 24-well chemotaxis chamber (Costar, Corning, cat# 3422) with a polycarbonate membrane of 8-μm pore size. Briefly, the cells were starved for 26 h in DMEM without FCS. Then, 100-μl starved MDA-MB231 cells in serum-free medium at a concentration of 3 × 10^5^ cells/ml were seeded on the upper compartments. Six hundred microliters of the fresh medium with 10% FCS was added into the bottom of the lower chamber. The prepared 24-well plates were respectively exposed to sham, 0.1-T RMF, or 0.4-T RMF. After 14 h of incubation, the nonmigrated cells on the upper surface of the membrane filter were removed, and the migrated cells attached to the lower surface of the filter were fixed with 4% formaldehyde solution and stained by 0.2% crystal purple solution. Migrated cells were quantitatively assessed by counting the number of cells under a light microscope. Relative migrated cell number was calculated by normalization to the sham control group.

The cell invasion assay was performed like the migration assay with slight modification. Briefly, the membrane filters of the 24-well chemotaxis chamber were coated with Matrigel (Becton Dickinson), and the invasion time was extended to 16 h.

### Rotating magnetic field setup

A pair of permanent magnets (rare-earth permanent magnet N42M) were equipped on a rotor with opposite poles facing up, which rotate at 4.2 Hz (the maximum speed of the instrument) clockwise. Both the magnets and the rotor were contained in a 44 cm × 41 cm × 52 cm (*L* × *W* × *H*) square box. In the sham control group, a pair of iron cubes were used instead. The distances between the magnets and the box surface were adjusted, and the maximum magnetic field intensities at locations of the cell culture plates were ~0.1 and ~0.4 T, respectively. Magnetic flux density distributions were measured by LakeShore 410 Gaussmeter (Lake Shore Cryotronics, Westerville, OH).

For each group, 2 mouse cages (each containing 6 mice) were placed on the surface of the box. The magnetic flux densities on the bottom of the mice cage are within 0.01- to 0.4-T range. Mouse cages of all 3 groups—the sham group (iron cube) and the 0.1-T and 0.4-T LF-RMF groups—were put on the instruments for 6 h (9AM-3PM)/d, 7 d/week, at 4.2 Hz for 4.5 months.

### Custom-designed cell incubator for RMF

We designed and constructed a set of biological sample incubation system (Fig. S1A), with accurate temperature, gas, and humidity control. A nonmagnetic stainless steel cylinder with a 300-mm outer diameter and a 200-mm inner diameter was the main part of the device, and the cylinder wall was hollow for flowing circulating water. A tube with a 10-mm inner diameter on the top cover of cylinder was used for flowing 5% CO_2_, and two 10-mm-inner-diameter tubes on both sides of cylinder were used for flowing circulating water. An electronic thermometer was used to monitor the temperature of sample chamber, which could be controlled by thermal conduction from the circulating temperature-controlled water, which flowed the cavity of cylinder wall. The temperature of the sample chamber can be controlled at 37 °C by adjusting the water temperature.

### Measuring F-actin in the central vs. peripheral areas

We used ImageJ software (ImageJ, RRID:SCR_003070) to measure the F-actin distribution in cells. Firstly, we drew 3 to 5 parallel lines perpendicular to the long axis in the cell, with both ends reach the cell edge. The length of the line segment was measured (*L*). Then, we drew 2 points at 1/4*L* inward from both ends of the line segment and connected the points to form a circle. The area inside of the circle is considered as the central area of the cell, and the area outside of the circle is considered as the peripheral area of the cell.

### Pyrene-actin assay

The actin mixture (rabbit skeletal muscle actin mixed with 10% pyrene-actin, Cytoskeleton, Cat. #AKL99 and #AP05) was diluted to 3.0 μM with G-buffer (5 mM Tris-HCl [pH 8.0], 0.2 mM CaCl_2_, 0.2 mM adenosine triphosphate [ATP], and 0.5 mM dithiothreitol) and left on ice for 1 h to depolymerize actin. The reactions were then centrifuged at 14,000 rpm at 4 °C for 30 min to remove residual nucleation centers. For polymerization assays, the supernatants were pipetted to a 96-well black opaque plate and measured in a fluorescent spectrophotometer to establish a baseline. Polymerization was induced by adding 10 × polymerization buffer (500 mM KCl, 20 mM MgCl_2_, and 10 mM ATP) to each well and mixing, followed by sham or LF-RMF treatment at room temperature for 5 min. The plate was returned to the spectrophotometer and was scanned every 30 s for 1 h. For depolymerization assays, supernatants were prepared and polymerized at room temperature for 1 h before they were centrifuged to pellet the polymerized actin (F-actin). F-actin was suspended in F-buffer (G-buffer + 1/10th the volume of 10× polymerization buffer) and added to a 96-well black plate. The baseline fluorescent was measured as described above. Depolymerization was induced by adding 2 μM Latrunculin A (Sigma-Aldrich, Cat. #L5163, RRID: SCR_004098), followed by a rotating magnetic field treatment at room temperature for 5 min. The plate was returned to the spectrophotometer and was scanned every 30 s for 1 h. The kinetics of polymerization and depolymerization were monitored using a SpectraMax i3x plate reader (Molecular Devices, San Jose, CA) with excitation and emission wavelengths of 360 and 420 nm, respectively.

### Animal assays

The animal protocols were approved by the ethical and humane committee of Hefei Institutes of Physical Science, Chinese Academy of Sciences. Specific-pathogen-free-grade 4-week-old female BALB/c (nu/nu) nude mice (RRID: IMSRAPB: 4790) were from Nanjing University-Nanjing Institute of Biomedicine. Mice were maintained in a sterile environment with light, humidity, and temperature control. After feeding for a week, experimental metastasis assays were performed by injecting 5 × 10^6^ MDA-MB231 cells/200 μl into mice tail veins. After intravenous injection of MDA-MB231 cells, we randomly divided 36 mice into 3 groups: sham, 0.1-T group, and 0.4-T group. For each group, *n* = 12. The mice were treated with sham or magnetic field on the second day after intravenous injection, from 9:00 AM to 3:00 PM every day, for 136 d. Weight and daily water and food consumption were measured every day. Mouse blood samples were collected from the eye orbit on the 45th day after intravenous injection for blood routine test. After 136 d, the mice were fed normally until their natural death (for the sham control group) or until 185 d. The lung and liver tissues were collected and processed for HE and Ki-67 staining.

### Rho protein activation assays

Amounts of RHOA and RHOA-guanosine triphosphate (GTP) were analyzed with the Rho Activation Assay Biochem Kit (Cytoskeleton, BK036). Cells were seeded at appropriate cell density (5 × 10^4^ cells/ml for MDA-MB231 cells and 1 × 10^5^ cells/ml for MCF-7 cells) in 100-mm dishes and grew for 3 d. LF-RMF treatment was performed when the cells were approximately 30% confluent. After LF-RMF treatment, all media was aspirated and the dishes were placed on ice immediately. Cells were rinsed with 10 ml of ice-cold PBS buffer to remove serum before they were lysed by 250 μl of ice-cold lysis buffer containing 1× Protease Inhibitor Cocktail (Roche) on ice for 5 min. Then, the cell lysates were harvested by a cell scraper. Cell lysates were transferred into the prelabeled sample tubes on ice and clarified by centrifugation at 10,000 × g, 4 °C for 1 min. Supernatants (20 μl) were used for protein concentration measurement by a protein concentration determination kit (P0009, Beyotime Biotechnology), and a 50 μg sample was used for Western blot quantitation of total RhoA in each sample. Equivalent amount of protein lysate (800 μg) was added to a 50 μg of rhotekin-RBD beads immediately for the pull-down assay. The reactions were incubated at 4 °C on a rotator or rocker for 1 h. Subsequently, the rhotekin-RBD beads were pelleted by centrifugation at 5,000 g at 4 °C for 1 min, and 90% of the supernatant was removed without disturbed the bead pellet. The beads were washed once with 500 μl of Wash Buffer and pelleted by centrifugation at 5,000 g at 4 °C for 3 min. Then, the supernatant was carefully removed without disturbing the bead pellet. Twenty microliters of 2 × Laemmli sample buffer was added to each tube to thoroughly resuspend the beads. The bead samples were boiled for 2 min and analyzed by SDS-PAGE and Western blot analysis. Eight hundred micrograms of supernatant was loaded with GTPγS as a positive control for the pull-down assay. For the negative control, supernatant was loaded with GDP in place of the GTPγS.

### Actin polymerization examined by TEM and DLS

The actin proteins (rabbit skeletal muscle actin, Cytoskeleton, Cat. #AKL99) were diluted to 3.0 μM with G-buffer (5 mM Tris-HCl [pH 8.0], 0.2 mM CaCl_2_, 0.2 mM ATP, and 0.5 mM dithiothreitol) before they were left on ice for 1 h and centrifuged at 14,000 rpm, 4 °C for 30 min to remove residual actin oligomers. Actin polymerization was induced by adding 10 × polymerization buffer (500 mM KCl, 20 mM MgCl_2_, and 10 mM ATP), followed by sham or LF-RMF treatment at room temperature for 10 min before they were processed for TEM or DLS measurement. For TEM experiment, the sample were added to a 200-mesh grid (20 s for ion sputtering) and incubated for 90 s. After the grid was dried, 1% uranyl acetate was added to the grid to allow incubation for 90 s. Then, the grid was stained by the dye solution 3 times before it was air-dried, and then the images were collected by a TEM (Talos F200X, FEI) operated at 200 kV. For the DLS experiment, the particle size distributions were examined by a Zetasizer Nano-ZSE Particle Analyzer (Malvern Instrument, Ltd., UK). All processes were carried out at room temperature.

### Statistical analysis

Statistical significance was determined by 2-tailed paired or unpaired Student *t* test. Statistical data are presented as means ± SEM (standard error of the mean) or SD (standard deviation). Sample size (*n*) and *P* value are specified in the text or figure legends. To reduced conscious or subconscious experimenter bias, we performed most data analysis in a blinded way by independent researchers. Their results are pooled together for statistical analysis.

## Data Availability

All data needed to evaluate the conclusions in the paper are present in the paper and/or the Supplementary Materials. Additional data related to this paper may be requested from the authors.
