# Supplementary material for: Intermittent F-actin Perturbations by Magnetic Fields Inhibit Breast Cancer Metastasis
Source: Research (Wash D C). 2023 Mar 15;6:0080. doi: 10.34133/research.0080 (PMC10017101; doi:10.34133/research.0080)
Supplement: Supplementary 1 — Supplementary Text Figs. S1 to S13. [file research.0080.f1.doc]

**Intermittent F-actin Perturbations by Magnetic Fields Inhibit Breast Cancer Metastasis**

Supplementary Materials

**Mice behavior tests**

***Balance beam experiment***

A self-made balance beam apparatus (1.5 cm × 80 cm, fig. S3A) was used to test the sense of balance and vestibular stimulated motor coordination of the mice. The beam was suspended 30 cm above the ground. Before experiment, the mice were placed in the box at the end of the beam for 2 min to familiarize themselves with the environment. Then they were given three trials to traverse the wooden beam to reach the box. In the first training, the mice climbed to the box from the endpoint of the beam, in the second training, the mice climbed from the midpoint, and in the last training, the mice climbed from the starting point. After the training, the mice were placed at the end of the balance beam to record the time they needed to reach the box. Performance was assessed between day 21 and 142 of RMF exposure and the experiments were repeated 16 times.

***Grip test***

We used a grip strength test to assess mice muscle strength. A self-made force testing apparatus (fig. S3B) was used, which the crossbar was 30 cm above the ground. Before the experiment, mice were trained three times so that they could grasp the crossbar with their forelimbs. During the experiment, mice were allowed to grab the crossbar with their forelimbs until the forelimbs released the crossbar, the grasping time was recorded. Performance was assessed between day 25 and 81 of RMF exposure and the experiment repeated 6 times.

***Open field test***

An open field test was used to assess the locomotor activity, exploratory and anxiety-like behaviors of the mice. Briefly, mice were gently placed in the square arena (50 × 50 × 30cm，fig. S3C) for 7 min before experiment. After habituating to the testing room, the mice were individually placed in the center of square arena and allowed to freely move for a 5-min period. The test sessions were recorded by a video camera placed 120 cm above the arena and analyzed using ANY-maze™ Video Tracking System (Stoelting Co. Wood Dale, IL, USA). Performance was assessed between the 20th and 141st days of RMF exposure and the experiment repeated 16 times.

**Hematoxylin and eosin (HE) staining and immunohistochemistry**

After the mice were sacrificed, their lung were fixed in 4% formaldehyde for 24 h before they were embedded in paraffin. Then the tissues were embedded in paraffin after formaldehyde removal, dehydration, cleaning and waxing. The paraffin-embedded specimens were then sectioned at 5 μm thickness.

For HE staining, the sections were stained with hematoxylin and eosin according the conventional HE staining procedures. For immunohistochemistry, the sections were incubated with hydrogen peroxide for 25 min to quench endogenous peroxidase activity before heated in an antigen retrieval solution. After blocking with 3% BSA for 30 min, the sections were incubated with antibodies against Ki67, PCNA, EGFR or Vimentin at 4 °C overnight.

**Supplementary Figures**


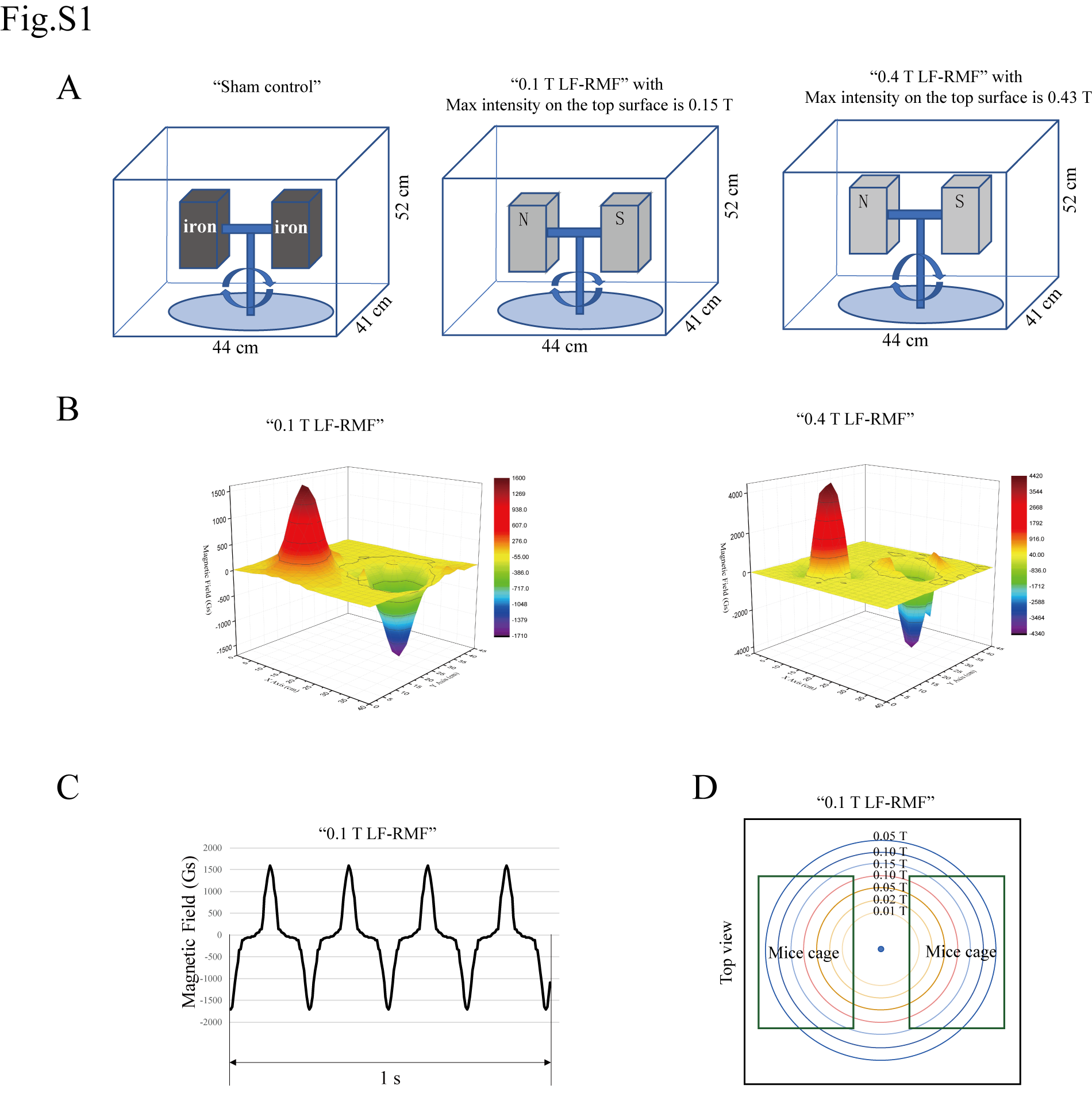


**Fig. S1. LF-RMF experimental setup. (A)** Schematic illustration of the sham control vs. the two LF-RMF instruments used in this study. The distances between the magnet in the two LF-RMF instruments are different, which generate differential magnetic field intensity on the surface of the box. **(B)** Magnetic flux density distribution on the surface of the two LF-RMF instruments. **(C)** Magnetic field induction as a function of time. **(D)** Magnetic field distribution on the surface of the 0.1 T LF-RMF instruments and the positions where the mice cages were placed.


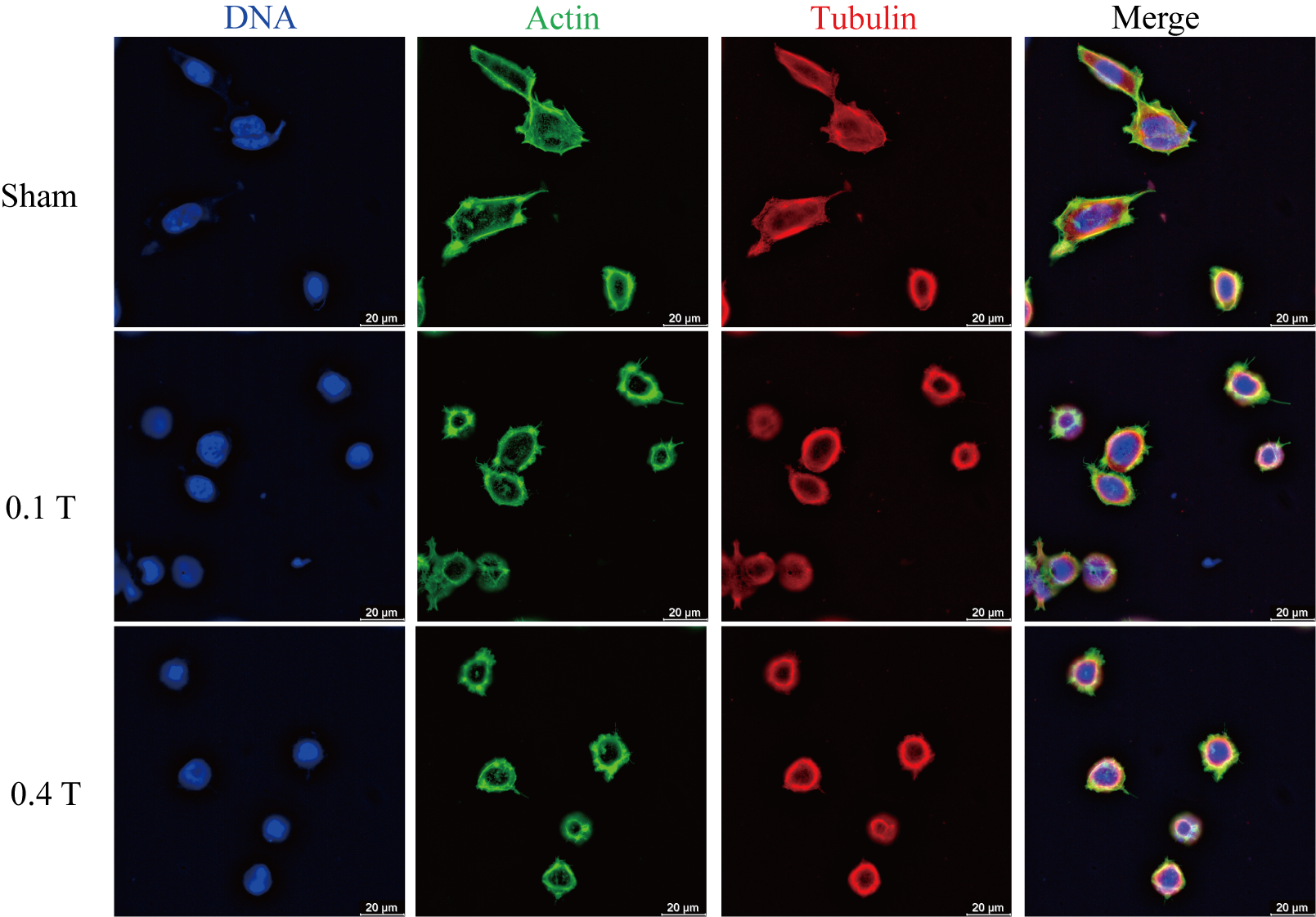


**Figure S2.** Immunofluorescent images of MCF-7 cells stained with phalloidin (green), anti-tubulin antibody (red) and DAPI (blue) after 6 h of RMF treatment. Cells were exposed to LF-RMF right after seeding to the plate. Scale bar: 20 μm.


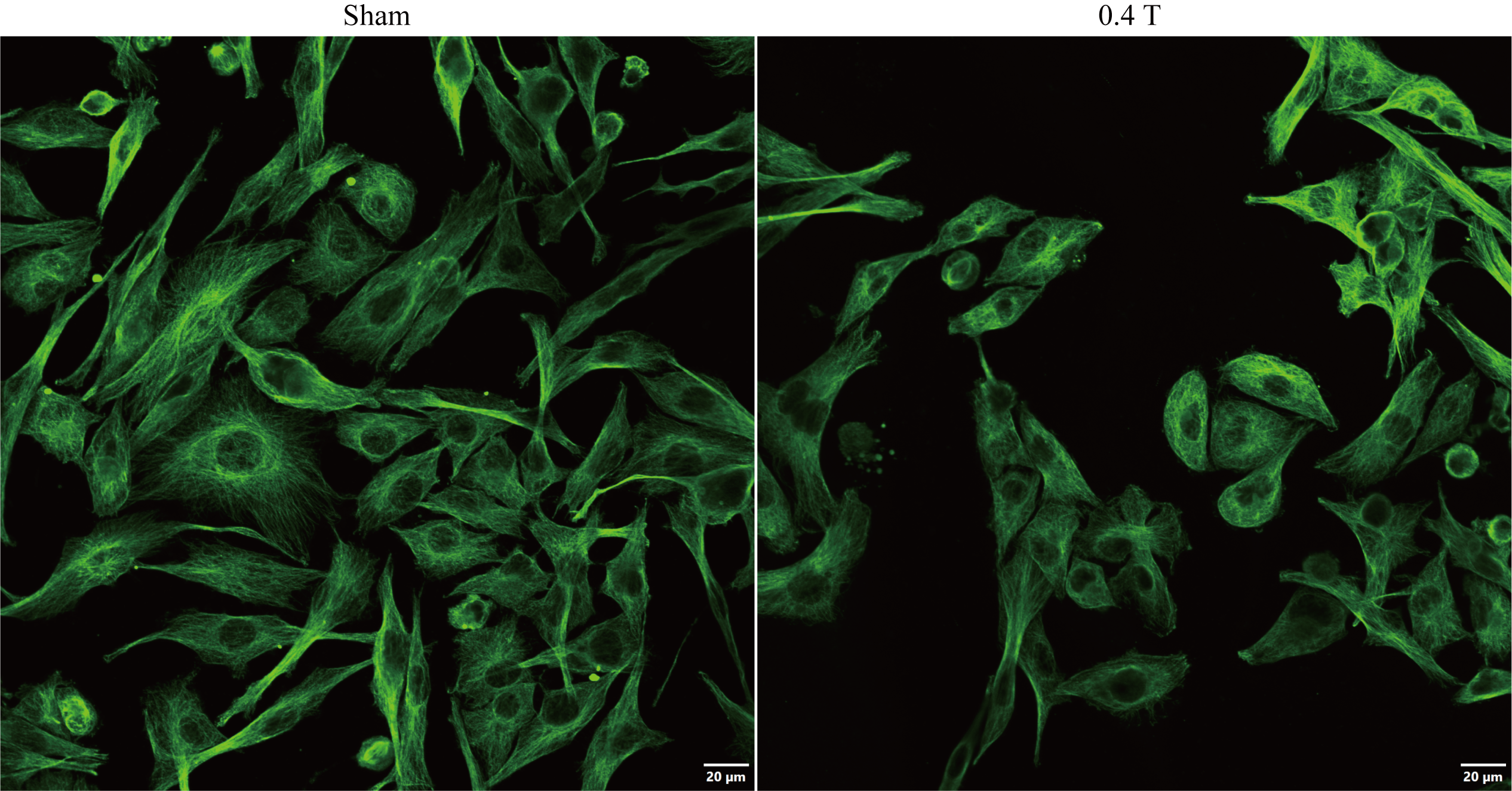


**Figure S3. Representative immunofluorescent images of microtubules in MDA-MB231 cells after LF-RMF treatment**. Cells were plated on coverslips overnight before they were exposed to 0.4 T LF-RMF for 30 hours, fixed and stained with anti-tubulin antibodies. Scale bar: 20 μm.


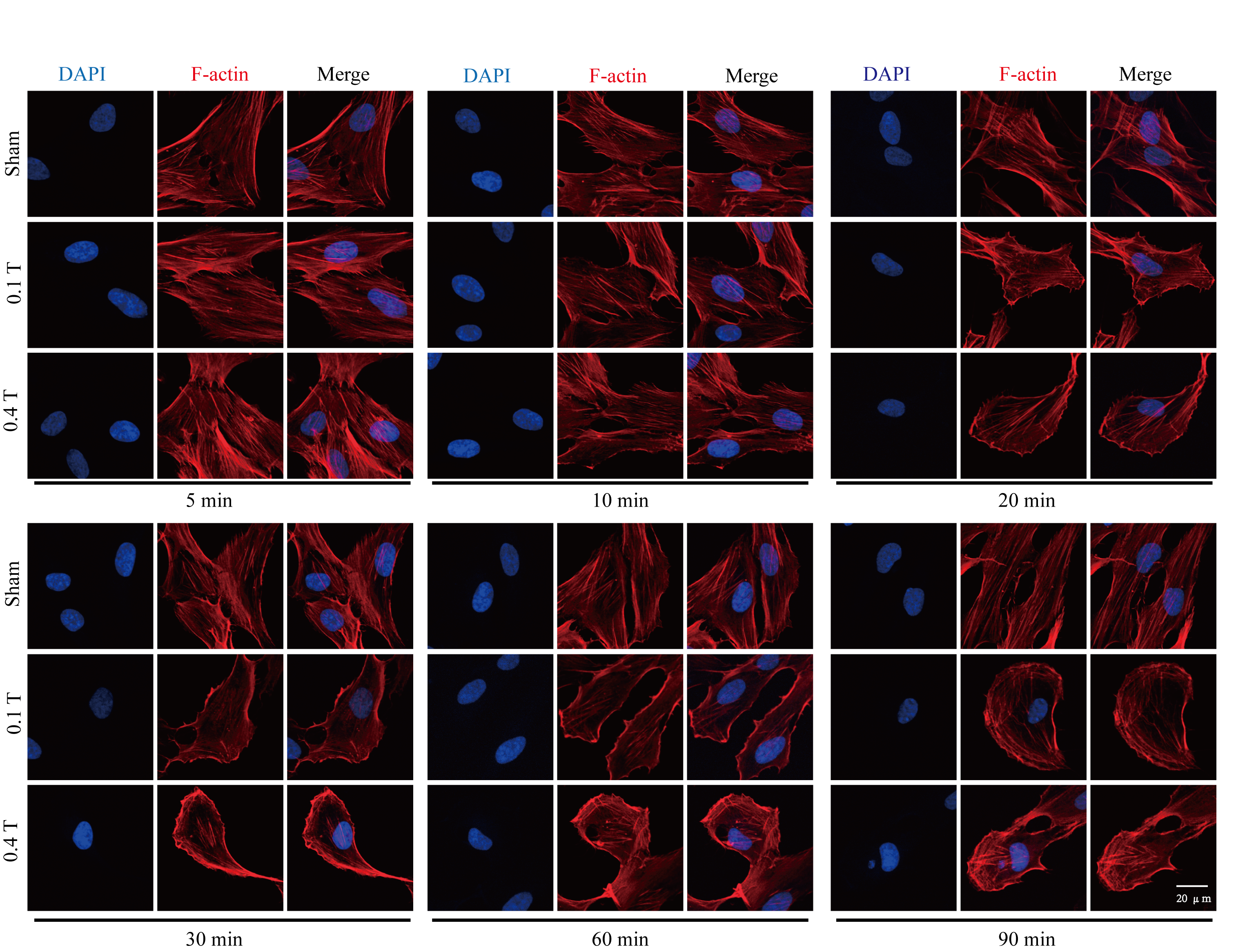


**Figure S4. Time-dependent effects of LF-RMFs on RPE1 cellular F-actin.** RPE1 cells were treated with sham, 0.1 T or 0.4 T RMFs for different timepoints. Cells were fixed and stained with phalloidin and DAPI. Scale bar: 20 μm.


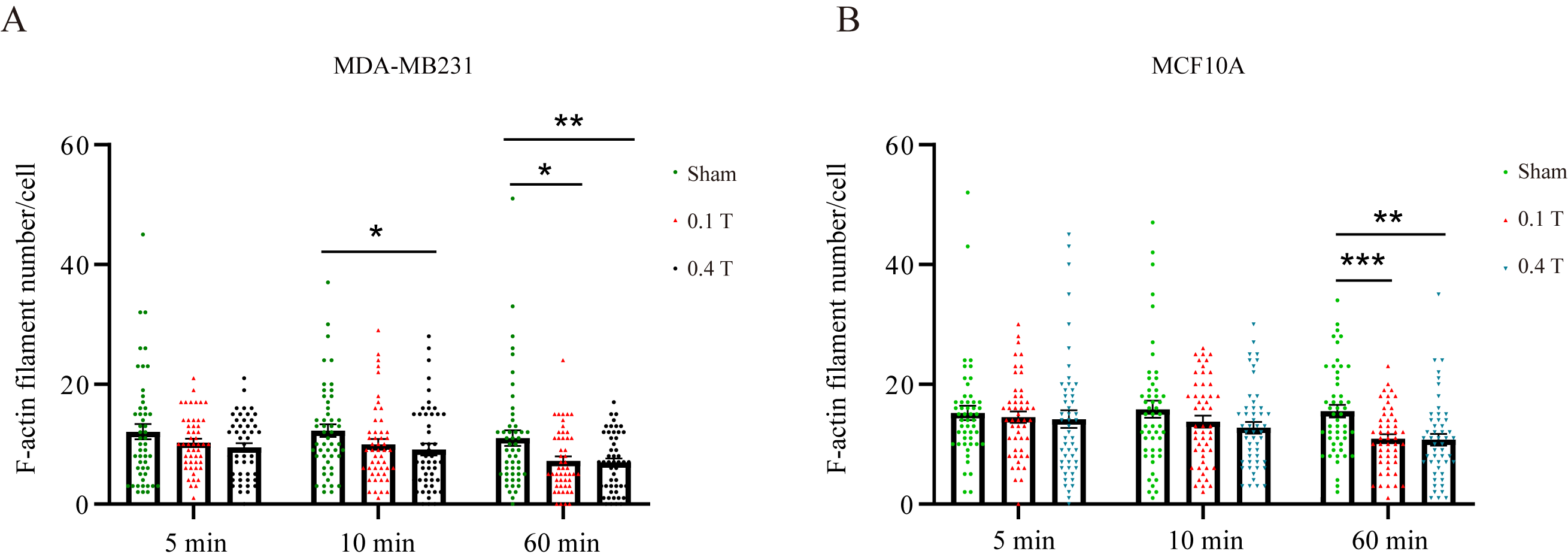


**Figure S5. Quantification of F-actin filament numbers in different cell lines treated with LF-RMFs for different time points. (A)** MDA-MB231 cells, **(B)** MCF10A cells were treated with sham, 0.1 T or 0.4 T LF-RMFs for 5, 10 or 60 minutes before they were stained with phalloidin and DAPI. 50 cells were quantified for each condition. Data are represented as means ± SEM. **P* < 0.05, ** *P* < 0.01, *** *P* < 0.005.


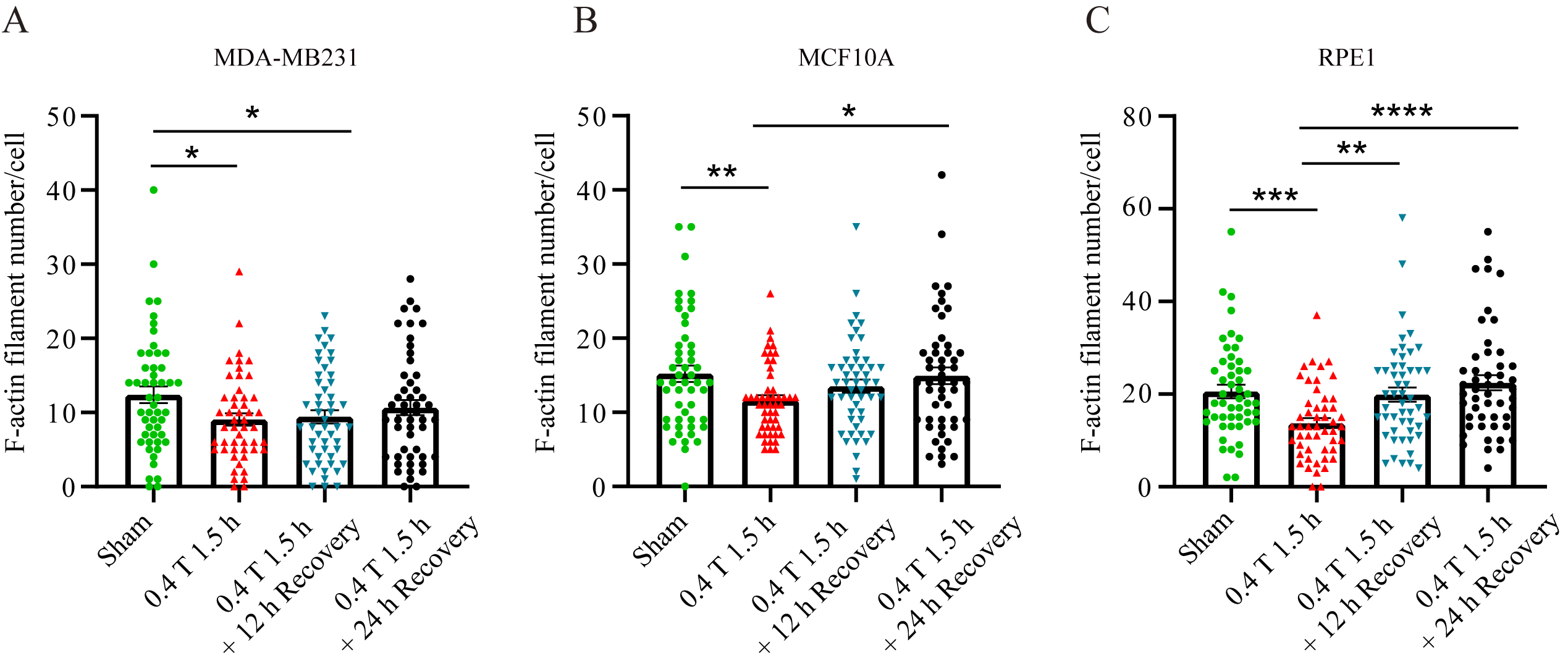


**Figure S6. Quantification of F-actin filament numbers in different cell lines treated with 0.4 T LF-RMF with or without recovery. (A)** MDA-MB231 cells, **(B)** MCF10A cells, **(C)** RPE1 cells were treated with sham or 0.4 T LF-RMFs for 1.5 hours followed by 0, 12-hour or 24-hour recovery (without LF-RMF treatment). 50 cells were quantified for each condition. Data are represented as means ± SEM. **P* < 0.05, ** *P* < 0.01, *** *P* < 0.005, **** *P* < 0.001.


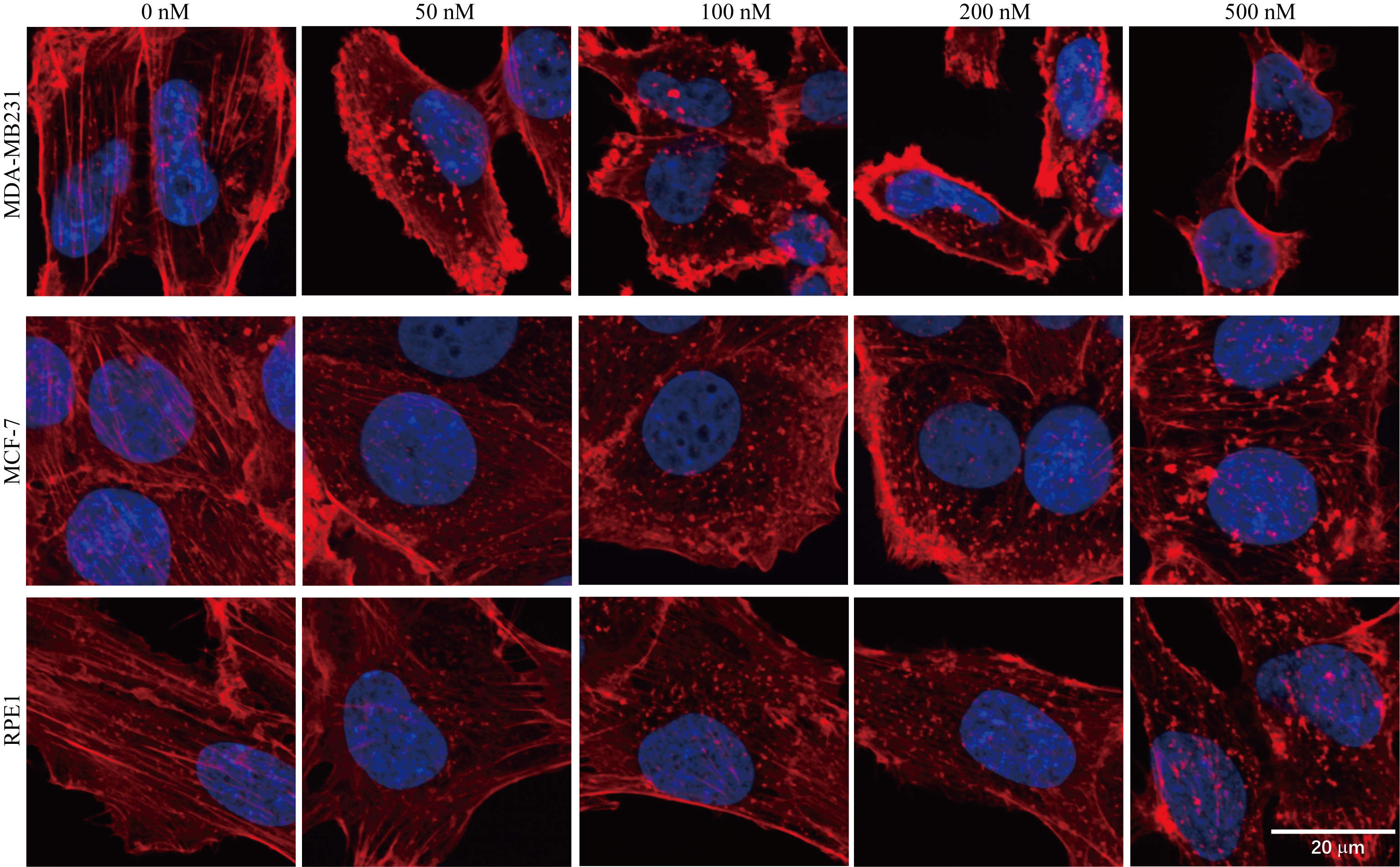


**Figure S7. The differential effects of cytochalasin D on F-actin in three different cell lines.** MDA-MB231**,** MCF-7 and RPE1 cells were treated with 0-500 nM cytochalasin D for 2 h before they were stained with phalloidin (red) and DAPI (blue). Scale bar: 20 μm.


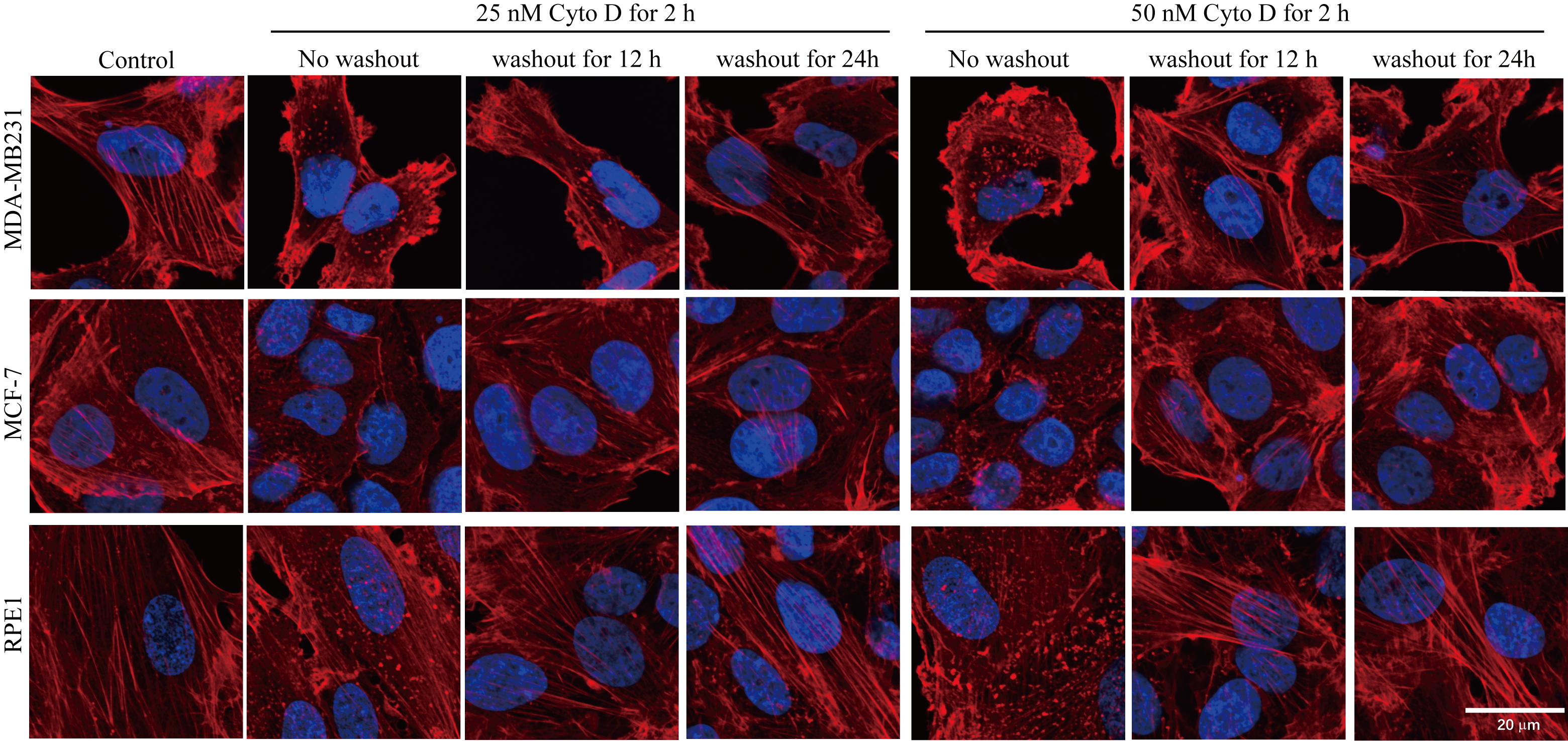


**Figure S8. The different sensitivity and recovery ability of F-actin in three different cell lines to cytochalasin D treatment and retrieval.** MDA-MB231, MCF-7 and RPE1 cells were treated with 25 or 50 nM cytochalasin D for 2 h, with or without additional washout to allow recovery for 12 or 24 h, before they were stained with phalloidin (red) and DAPI (blue). Scale bar: 20 μm.


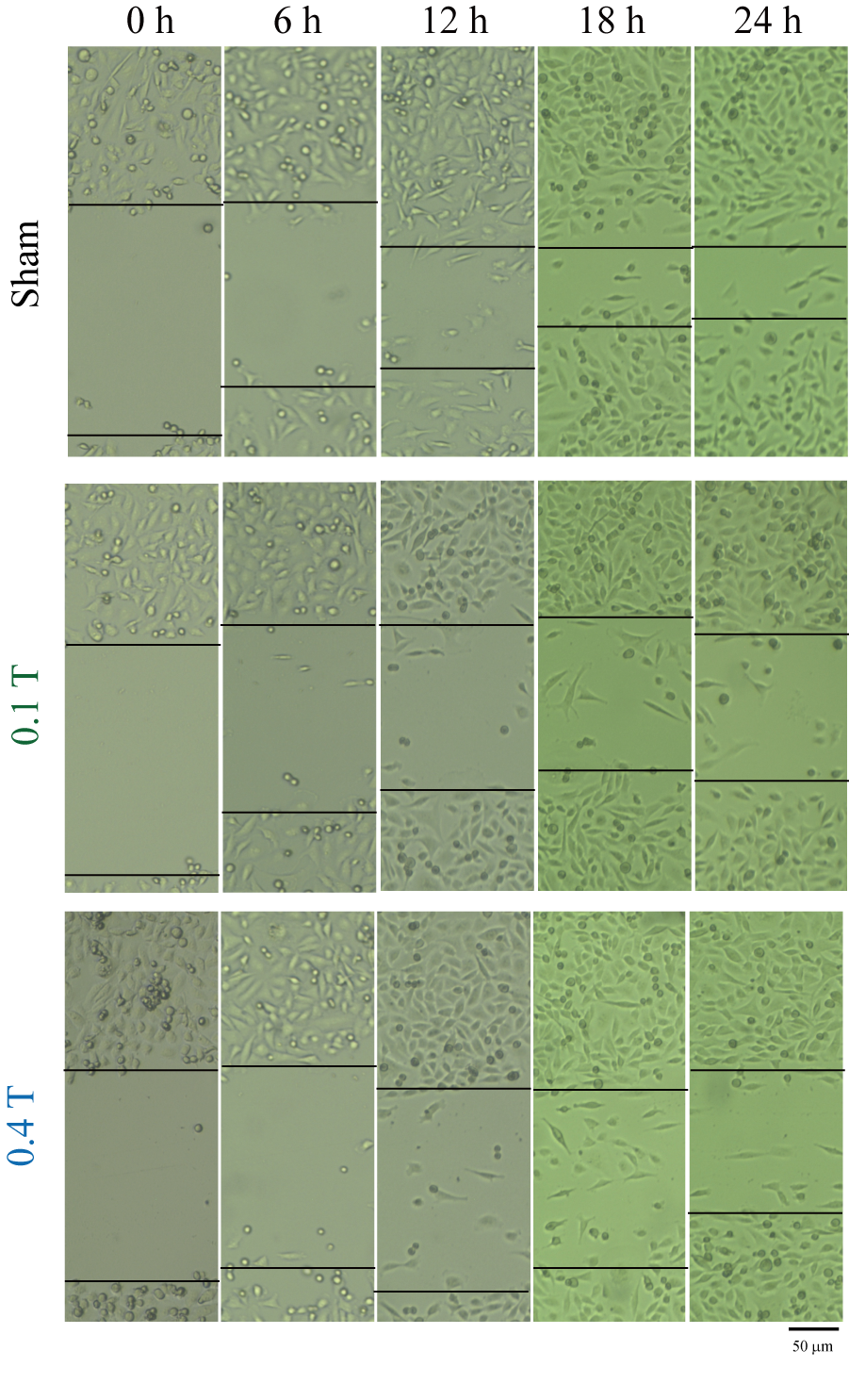


**Figure S9. Wound healing migration assay shows that LF-RMFs can decrease breast cancer MDA-MB231 cell migration.**


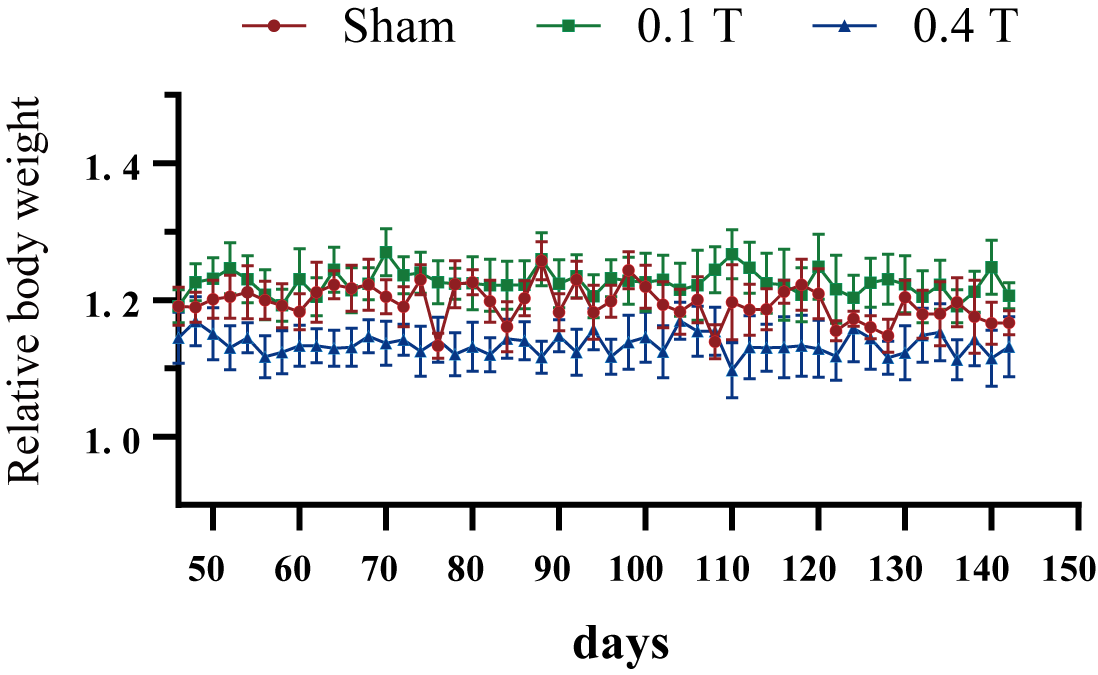


**Figure S10. Relative body weight of mice in different groups.** Data are represented as means ±SEM.


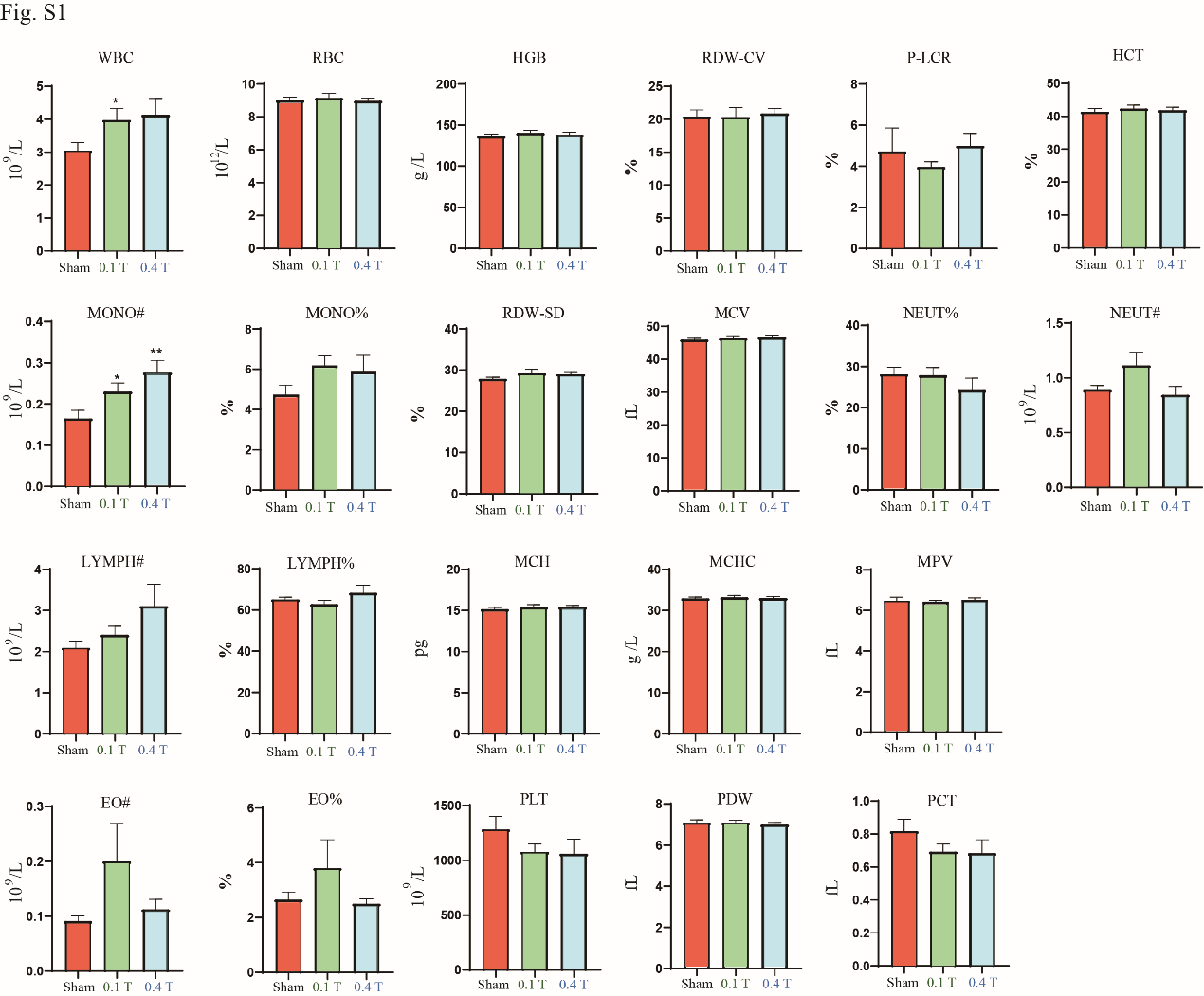


**Figure S11. Blood routine of LF-RMF-treated MDA-MB231 bearing mice.** Comparisons were made between each sham control and the corresponding LF-RMF groups. Data are represented as means ±SEM. **P* < 0.05, ***P* < 0.01.


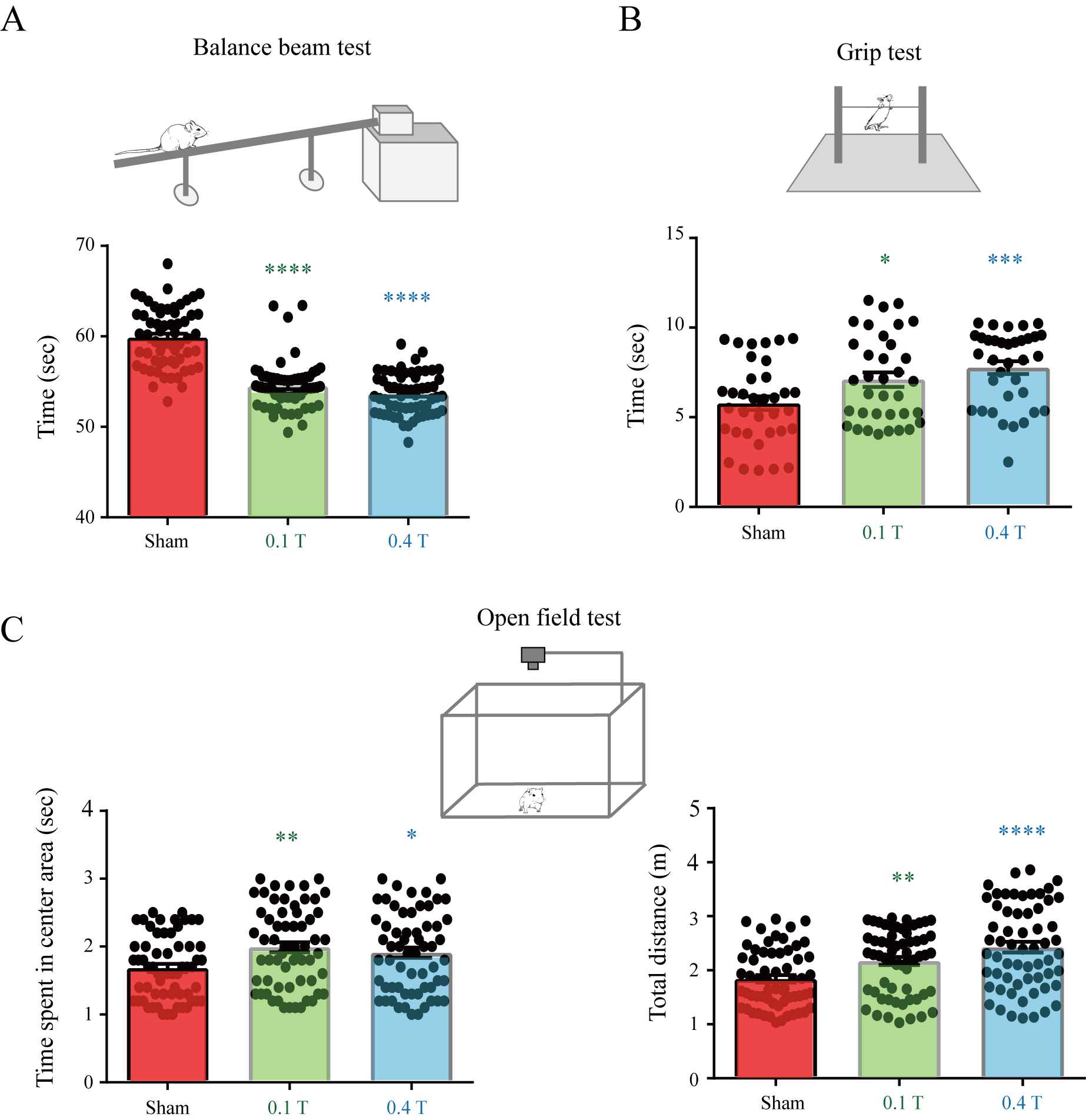


**Figure S12. LF-RMFs improve MDA-MB231 bearing mice motor coordination, muscular strength and exploratory activity. (A)** The traverse time in Balance beam test. **(B)** The grip time in grip test. **(C)** The time spent in the center area and total travel distance in open field test. Data are represented as means ± SEM. **P* < 0.05, ***P* < 0.01, *** *P* < 0.005, **** *P* < 0.001.


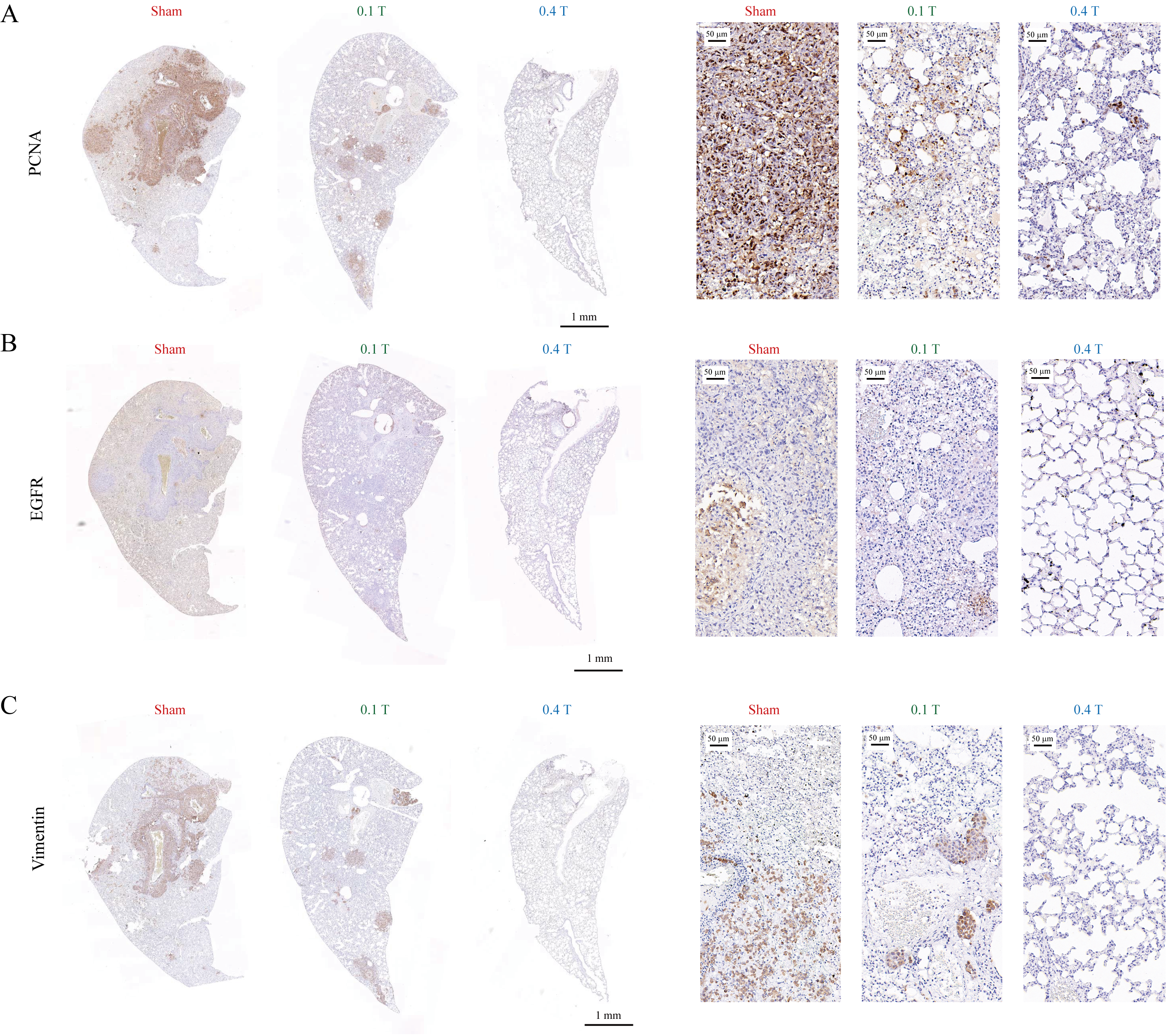


**Figure S13.** LF-RMF-treated mice have much reduced proliferating cancer modules in their lung tissues. Representative immunohistochemistry images of **(A)** PCNA, **(B)** EGFR, **(C)** Vimentin in the lung tissues of sham control, 0.1 T LF-RMF and 0.4 T LF-RMF treated MDA-MB231 bearing mice.
